# The Behavioral Space of Zebrafish Locomotion and Its Neural Network Analog

**DOI:** 10.1371/journal.pone.0128668

**Published:** 2015-07-01

**Authors:** Kiran Girdhar, Martin Gruebele, Yann R. Chemla

**Affiliations:** 1 Center for Biophysics and Computational Biology, University of Illinois, Urbana, IL, 61801, United States of America; 2 Department of Physics, Center for the Physics of Living Cells, University of Illinois, Urbana, IL, 61801, United States of America; 3 Department of Chemistry, University of Illinois, Urbana, 61801, United States of America; University Zürich, SWITZERLAND

## Abstract

How simple is the underlying control mechanism for the complex locomotion of vertebrates? We explore this question for the swimming behavior of zebrafish larvae. A parameter-independent method, similar to that used in studies of worms and flies, is applied to analyze swimming movies of fish. The motion itself yields a natural set of fish "eigenshapes" as coordinates, rather than the experimenter imposing a choice of coordinates. Three eigenshape coordinates are sufficient to construct a quantitative "postural space" that captures >96% of the observed zebrafish locomotion. Viewed in postural space, swim bouts are manifested as trajectories consisting of cycles of shapes repeated in succession. To classify behavioral patterns quantitatively and to understand behavioral variations among an ensemble of fish, we construct a "behavioral space" using multi-dimensional scaling (MDS). This method turns each cycle of a trajectory into a single point in behavioral space, and clusters points based on behavioral similarity. Clustering analysis reveals three known behavioral patterns—scoots, turns, rests—but shows that these do not represent discrete states, but rather extremes of a continuum. The behavioral space not only classifies fish by their behavior but also distinguishes fish by age. With the insight into fish behavior from postural space and behavioral space, we construct a two-channel neural network model for fish locomotion, which produces strikingly similar postural space and behavioral space dynamics compared to real zebrafish.

## Introduction

Behavior is a direct reflection of neural activity and its modulation by external stimuli. Many tools are available to study behavior—for example, electrophysiological techniques to probe neural circuitry [1], non-invasive behavioral assays that simply record the motion of an animal [2–5], and genetic manipulation that perturb the system [6, 7]. Here we analyze the free swimming behavior of zebrafish, and ask: Can the motion itself tell us which coordinates are needed to describe behavior quantitatively, how to describe the relationship among behaviors without *a priori* classification, and ultimately how to construct a neural model for the behavior and check its success?

The zebrafish (*Danio rerio*) is a tropical freshwater fish native to the Himalayan region and a model organism for studying behavior [8]. Zebrafish larvae 7–10 days post fertilization (dpf) exhibit a variety of swimming behaviors, yet possess only a small number (~200) of locomotor neurons that project from the brainstem into the spinal cord [9]. Despite its simplicity, this vertebrate neural system is capable of complex behaviors such as spontaneous scooting and turning, prey tracking, escape response in reaction to different kinds of stimuli (acoustic and tactile) [9, 10] and optomotor response [11]. All of these behaviors must arise from simple neural control patterns. The wide range of behavioral patterns, ease of imaging, yet simplicity of the larval brain and motor neurons thus make zebrafish ideal for quantifying and modeling behavior.

Extensive studies have categorized the behavioral patterns of zebrafish [10, 12]. A common approach [9] to study their swimming is to classify the behavior according to predetermined sets of parameters related to fish shape and swimming direction: change in head angle, angular velocity, and bend duration, to name a few. By applying boundary criteria to these predetermined parameters, the zebrafish free swimming, escape response (response to external stimuli like a pressure pulse, acoustic or tactile), and prey tracking behaviors [10, 13–15] are classified into discrete patterns: the routine turn (or R-turn), scoot, C-turn, J-turn, etc. For example, R-turns and scoots have been defined as changes in the heading direction by >30–40° and <30–40°, respectively [9, 10]. A limitation of this approach is that it relies on *a priori* parameters and imposed criteria to define the organism’s behavioral repertoire. The question arises whether the animal itself can provide a “parameter-free” basis for the description of its motions.

Recent work [16, 17] has drawn on methods in artificial intelligence to analyze quantitatively the different set of behaviors an entity can perform. A study of the invertebrate *C*. *elegans* by Stephens *et al*. [18] provided a powerful method to quantify worm locomotion in terms of the worm’s shapes. Its movements can be represented as a simple cyclic trajectory in a “postural space”, which consists of a few carefully chosen coordinates to represent the multiple shapes the organism cycles through. Other studies [19–22] have shown that the complexity and dimensionality of behaviors can be studied by embedding such trajectories in a low-dimensional “behavioral space.”

**Fig 1:**
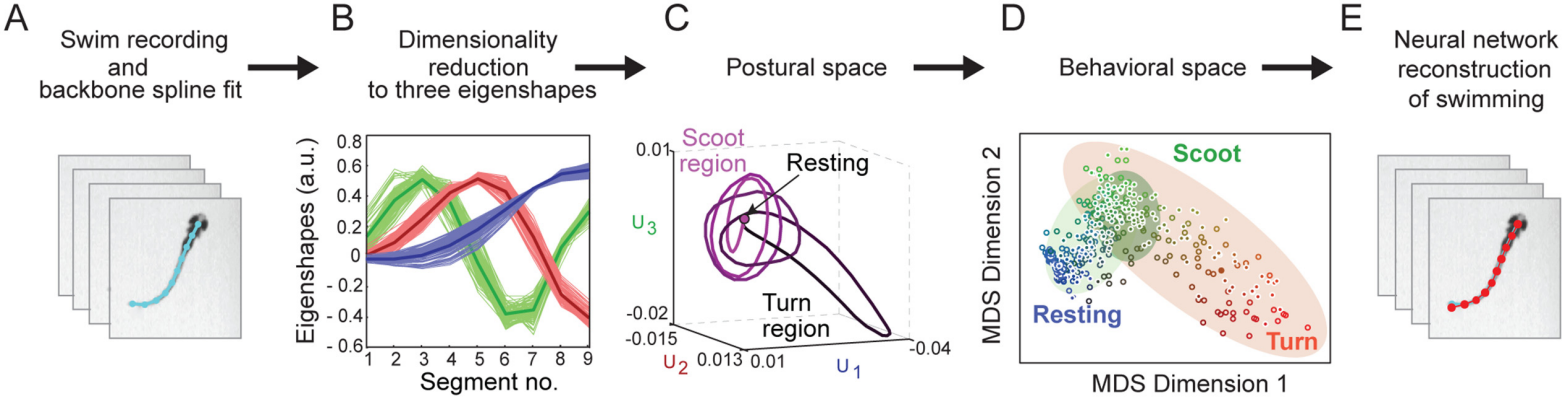
Workflow from swim observation to neuro-kinematic model. (A) Snapshots of a larval zebrafish free swimming movie in which the fish backbone is fitted to a 10-point spline. (B) A linear combination of three “eigenshapes” accurately reconstructs the backbone shapes of the zebrafish. (C) A swimming bout is represented as a trajectory in the “postural space” spanned by the three eigenshapes. (D) A “behavioral space” generated by multi-dimensional scaling reduces each cycle of a trajectory to a point, and clusters them by their similarity. (E) A 2-channel neuro-kinematic model is constructed based on the observed behavioral patterns and evaluated using the same work flow.

In the present work, we apply similar approaches to zebrafish to understand the behavioral patterns in their free swimming. The key steps are summarized with actual data in Fig 1. (A) We film individual fish in a non-invasive behavioral assay. (B) A parameter-free analysis yields three orthogonal “eigenshapes” that reconstruct accurately zebrafish postures as a function of time. (C) In the resulting three-dimensional “postural space” the temporal sequence of shapes appears as a trajectory. (D) To quantify the range of behaviors spanned by these trajectories, we compare multiple swim bouts and animals of different ages and measure the similarity of their trajectories to one another. Each cycle of a trajectory is represented in a “behavioral space” as a single point in a neighborhood with others displaying similar patterns. This behavioral space separates fish behaviors by behavioral pattern and by age. A clustering analysis shows that three behavioral patterns can be identified, corresponding to scoots, turns, and rests. However, the probability distribution of behaviors shows that while scoots and rests are discrete maxima, turns are represented by a wing on the scoot maximum. (E) We construct a two-channel neural network/kinematics model based on *xenopus* swimming [23] that mimics zebrafish swimming robustly. Thus we close the loop from initial observation to neuro-kinematic simulation of zebrafish behavior. The analysis of worms [18], flies [19] and zebrafish shows that intrinsic coordinates, behavioral space, and dimensionality reduction can be powerful tools to quantify and compare animal behavior without *a priori* classification, and to inform construction of neural simulations for animal motion.

## Results

### Zebrafish backbone shapes while free swimming are described by just a few orthonormal basis functions

**Fig 2:**
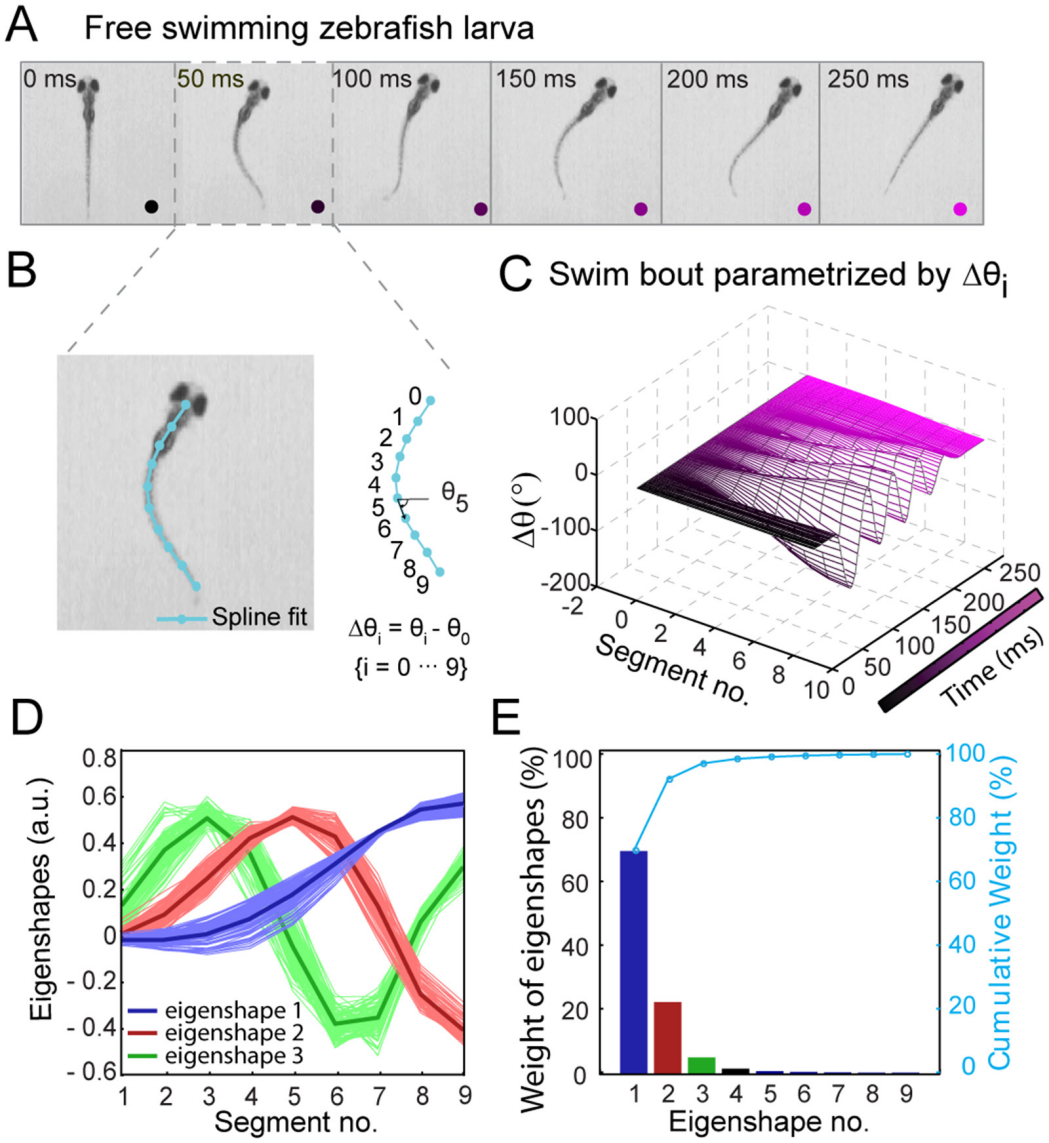
Decomposition of free swimming zebrafish larval backbone shapes into orthonormal basis. (A) Still images of a swim bout from a representative movie of free swimming zebrafish larva, recorded at 500 fps. (B) Spline fit of fish backbone (cyan). Tangent vectors (black arrow) at 10 evenly spaced segments along the backbone from head (*s*_0_ = 0) to tail (*s*_9_ = 9) point along a direction *θ*(*s*_*j*_). (C) Swim bout from A parameterized by Δ*θ*(*s*_*j*_,*t*_*i*_) = *θ*(*s*_*j*_,*t*_*i*_)–*θ*(0,*t*_*i*_). (D) Singular value decomposition of Δ*θ* into eigenshapes *V*_*k*_(*s*_*j*_) (*j* = 1, 2… 9), the first three of which are plotted (red, blue, and green, respectively). The light colors correspond to eigenshapes determined from analyzing individual fish from a population, and dark colors are the collective eigenshapes from analyzing the entire population at once. (E) Bar plot of % weights ($S_{kk}/\sum_{k\prime =1}^{10}S_{k\prime k\prime }$, where *S*_*kk*_ are the singular values) of each eigenfunction *V*_*k*_(*s*_*j*_). The right axis in cyan shows the cumulative contribution of each eigenshape. The first three eigenshapes contribute 96% of the total variance in Δ*θ*.

How can one quantify the free swimming behavior of zebrafish without *a priori* criteria to classify the observed behavior? To answer this question, we applied singular value decomposition (SVD) [24] to the sequence of zebrafish backbone shapes as they evolve during swimming. SVD is a parameter-free linear algebra technique that, in our application, reduces all shapes to a small number of linearly independent “eigenshapes.” We recorded video of individual larvae swimming freely in quasi-2D in a petri dish at a rate of 500 frames per second (fps) using a high-speed camera (S1 Movie and Materials and Methods). Each video typically had 4–5 swimming episodes of duration ~250 ms each, separated by pauses. The video was divided into movies of individual swimming bouts which were analyzed separately. The movies consisted of a sequence of *m* frames at times *t*_*i*_. Fig 2A shows still images from a representative swimming bout (see Fig 2A).

Next, we applied background subtraction algorithms and threshold procedures to the movies to extract the backbone shape of each fish as a sequence in time (see S1A–S1F Fig). The backbone shape in each frame was fitted to a cubic spline (see Materials and Methods and S1G Fig). We sampled the spline at 2 to 100 points (S2 Fig), and found that *n* = 10 points were sufficient to converge the spline to camera pixel resolution of the backbone. This procedure allows an accurate but very compact representation of the backbone trajectories, enabling low-dimensional visualization. As shown in Fig 2B, the 10-points spline curve was converted into a one-dimensional array of 10 spine angles *θ*(*s*_*j*_,*t*_*i*_), measured along the normalized arc lengths of the fish from head (*s*_0_ = 0) to tail (*s*_9_ = 1). All spine angles are given relative to the head angle, Δ*θ*(*s*_*j*_,*t*_*i*_) = *θ*(*s*_*j*_,*t*_*i*_)–*θ*(*s*_0_,*t*_*i*_).

Δ*θ*(*s*_*j*_,*t*_*i*_)is an *m* × *n* matrix in which each row is the spine angles from head to tail, and successive rows down the matrix represent snapshots at different times. In Fig 2C, Δ*θ* = 0 at the head and swings out to maximum values towards the tip of the tail. The matrix Δ*θ*(*s*_*j*_,*t*_*i*_) was then decomposed into a set of *n* = 10 linearly independent, orthonormal basis functions *V*_*k*_ by SVD [24], as given by the relation

$$\Delta \theta \left(s_{j},t_{i}\right)\;=\sum_{k=1}^{n}U_{k}(t_{i})S_{kk}V_{k}(s_{j})$$ (1)

The basis functions represent eigenshapes of the zebrafish backbone (Fig 2D **)**. Their linear combination reconstructs the spline-fitted backbone shapes exactly, according to Eq (1). Each *U*_*k*_(*t*_*i*_) represents the amplitude of the *k*-th basis function *V*_*k*_(*s*_*j*_) at each time point *t*_*i*_. *S*_*kk*_ is an *n* × *n* diagonal matrix of singular values. *S*_*kk*_ is conventionally normalized to 1, such that each singular value represents the fractional contribution a basis function makes to the overall swimming behavior. The basis functions are sorted from most important (largest singular value; *k* = 1) to least important (smallest singular value; *k* = *n*). The key element of this analysis is that many of the singular values in the matrix *S*_*kk*_ may be small, and thus many basis functions can be left out of the sum in Eq (1) with negligible effect. As shown in Fig 2E, performing SVD on all movies (*N* = 115) from a population of 20 fish reveals that 96% of the variation in Δ*θ* is accounted for by the first three eigenshapes only, i.e. taking the summation in Eq (1) only up to *n* = 3. The residual error was ~7% between the spine angles Δ*θ*(*s*_*j*_,*t*_*i*_) *and* reconstructed spine angles Δ*θ* ^*r*^(*s*_*j*_,*t*_*i*_) using the first three eigenshapes (see S3 Fig **)**.

Plotting the three eigenshapes (light blue, red, and green in Fig 2D) from individually analyzed movies shows that the basis functions were similar across the population of fish. The collective eigenshapes shown in dark colors (dark blue, red, and green) were obtained by analyzing all movies together. Translated into real space (S4 Fig **)**, the three basis functions correspond to zebrafish shapes with the following features: a single bend at the midpoint (blue), bends at the head and at the tail (red), and bends at the head, midpoint, and tail (green).

### Stereotyped behaviors are visualized in a quantitative low-dimensional postural space

**Fig 3:**
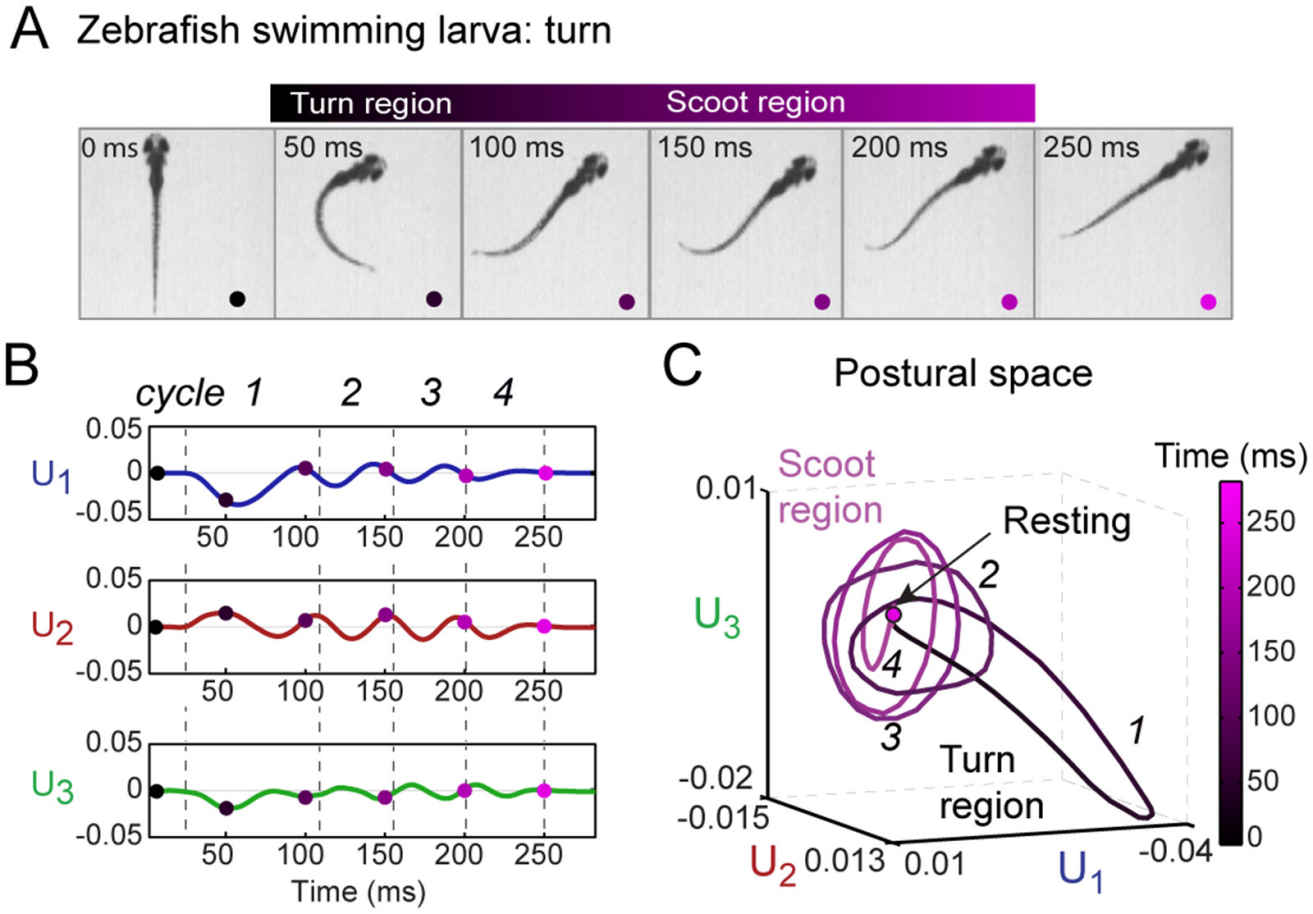
Representation of free swimming zebrafish in low-dimensional postural space. (A) Still images of a representative turning bout during free swimming. As discussed in the text, it is convenient to divide the bout into a “turn” region (*t* = 50–100 ms) followed by a “scoot” region (100–250 ms). (B) Plot of the amplitudes *U*_1_(*t*), *U*_2_(*t*), and *U*_3_(*t*) of the three collective eigenshapes corresponding to the movie in A. Each amplitude undergoes multiple oscillation cycles before returning to zero. The regions marked by dashed lines and labeled as cycles (1–4) in *U*_1_(*t*), *U*_2_(*t*), and *U*_3_(*t*) are obtained from the oscillation cycles in *U*_1_(*t*). The colored dots mark time points corresponding to the still images in A. (C) Representation of a turn bout in postural space. The three-dimensional coordinates of the trajectory are the amplitudes *U*_1_(*t*), *U*_2_(*t*), and *U*_3_(*t*) in B. In this space, the bout involves a turn region (*t* = 50–100 ms), represented as a bent ellipse (cycle 1), followed by a scoot region (*t* = 100–250 ms) represented as multiple cycles (2–4) along the flat ellipses, and a final return to the rest behavior. Throughout, time (0–250 ms) is represented by the black—magenta colormap. An analysis of a representative scooting bout is shown in S5 Fig.

Based on this analysis method, any zebrafish free swimming bout can be represented as a trajectory in the low-dimensional postural space spanned by the three collective eigenshapes described above. Fig 3 shows an example of a zebrafish turning bout and how it can be visualized as a trajectory in the postural space (S5 Fig shows a corresponding scooting bout). At each time point *t*_*i*_ of the movie, the zebrafish backbone shape is represented by a set of three amplitudes {*U*_*k*_(*t*_*i*_)} with *k* = 1, 2 and 3. Fig 3B plots these three amplitudes *U*_*k*_ vs. time, and in Fig 3C the three amplitudes define the coordinates of this fish’s trajectory in the three-dimensional postural space. Represented in this manner, a swimming bout appears as a sequence of cycles of shapes. For this particular example the swim bout has four cycles as shown by dashed lines in Fig 3B that explore different regions of the postural space in Fig 3C.

Previous studies have categorized free swimming of zebrafish into two behavioral states, the “R-turn” and “scoot”, based on a limiting angle of the backbone [9, 10]. Turns correspond to a major change in swimming direction and are specifically defined as a swimming bout at the start of which the tail makes an angle >30–40° relative to the head. In contrast, scoots are swimming bouts with no significant change in direction and are defined by the tail making an angle relative to the head <30–40° [9, 10]. As shown in S5 Fig, a scoot bout is represented in postural space by oscillation cycles predominantly in *U*_2_ and *U*_3_, returning to a fixed point corresponding to a “resting” fish. In contrast, the turn bout in Fig 3C takes an excursion along the *U*_1_–*U*_3_ plane lasting between one half and one whole oscillation of cycle 1, before shifting to cycles 2–4 in *U*_2_ and *U*_3_ similar to those in the scoot trajectory (see cycles 1–5 in S5C Fig) and eventually returning to the resting behavior. (See S2 and S3 Movies) for demonstrations of a turn and a scoot bout in 3-D postural space, respectively.) The sign of *U*_1_ corresponds to the direction of the turn, with *U*_1_ < 0 corresponding to a right turn and *U*_1_ > 0 to a left turn, and we consider these cases to be mirror images of the same behavior. To distinguish the different cycles of a trajectory in postural space, we found it useful to name the excursion in the *U*_1_–*U*_3_ plane “turn region”, the oscillation cycles in the *U*_2_–*U*_3_ plane “scoot region” and finally decaying to zero amplitude as “resting region.” Thus a swimming bout can progress through these regions in postural space in succession. Time is color coded from black to magenta on the trajectory in Fig 3C, corresponding to the dots in Fig 3A.

### Multi-dimensional scaling of trajectories from postural space reveals the behavioral space of zebrafish free swimming

How does this approach extend to an ensemble of swimming bouts? To determine if multiple trajectories can be similarly categorized, we compared trajectories from different fish, normalizing their time axes by matching the duration of the first oscillation period in *U*_*1*_ *(t)* termed “cycle 1;”(see S1 File and S6A Fig). The histogram of normalized time was in the range of 15–33 ms of data set as shown in S6B Fig We also normalized the sign of the trajectory amplitudes, so that handedness is ignored in our analysis. Our data set included both younger (7–8 dpf) and older larvae (9–10 dpf) to determine if factors such as age alter free swimming behavior significantly.

**Fig 4:**
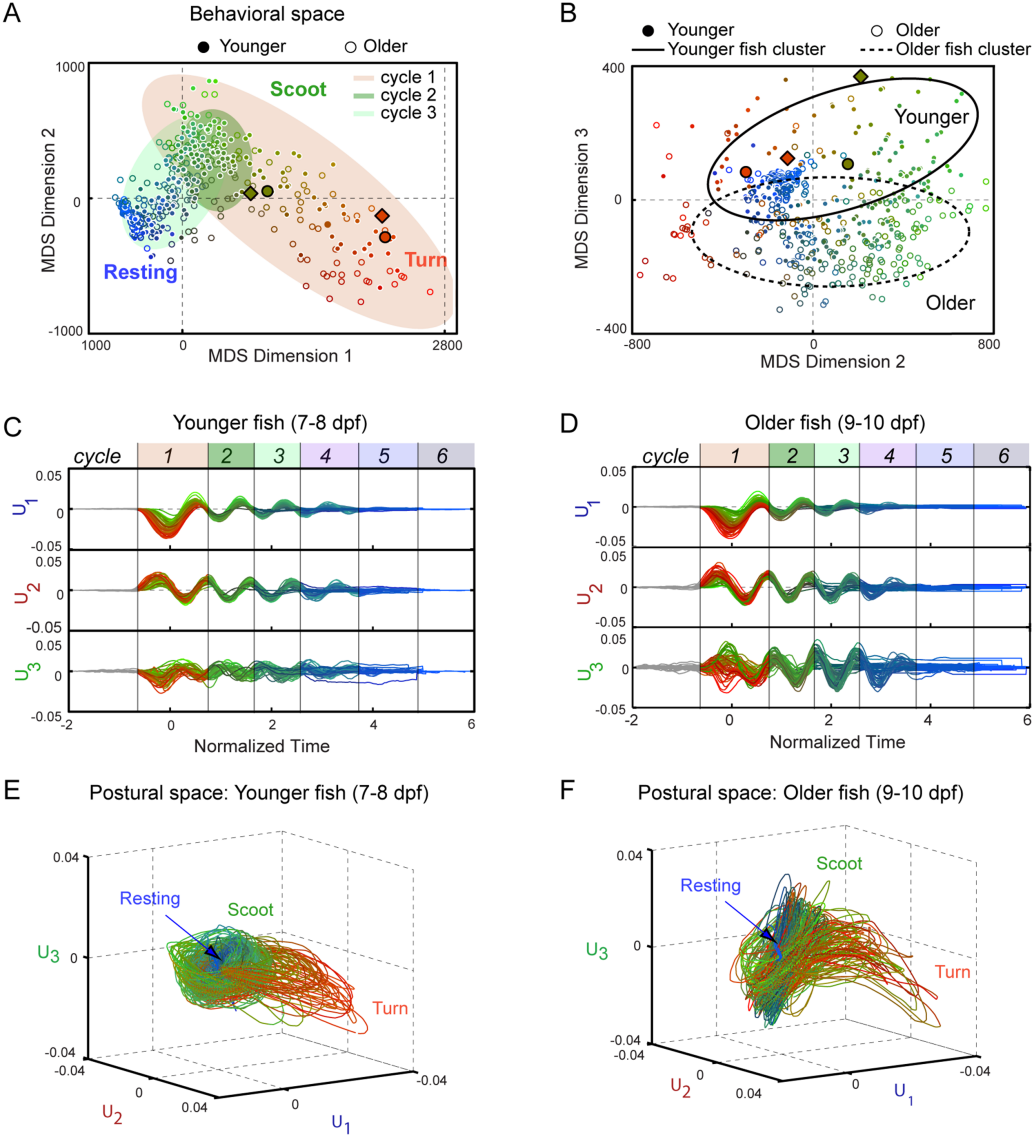
Behavioral space of free swimming zebrafish. (A–B) Ensemble of swim bout trajectories (*N* = 115) embedded by multi-dimensional scaling (MDS) into low-dimensional “behavioral” space. Each cycle of every trajectory is represented by a single point (filled circles for younger larvae (*N* = 8; 7–8 dpf), open circles for older larvae (*N* = 12; 9–10 dpf)). The distances between points reflect their behavioral similarities/differences. MDS dimensions 1 and 2 (A) reveal behavioral regions corresponding to turns (red points), scoots (green) and rests (blue). The shaded ellipses demarcate the points from cycles 1–3 of the trajectory (orange, light green, green, respectively. S8A Fig displays the cycles individually). MDS dimensions 2 and 3 (B) reveal differences with zebrafish age (solid ellipse for younger larvae; dotted ellipse for older larvae). The ellipses were determined using PCA analysis of the behavioral space in MDS dimension 1 and dimension 2 for each cycle in (A) and MDS dimension 2 and dimension 3 for cycle 1 in (B). The principal axes of the ellipses are the singular values from PCA. Simulated trajectories from the neuro-kinematic model in Figs 5 and 6 and its training data are shown in diamonds and circles with black outline, respectively. (C) Amplitudes *U*_1_, *U*_2_, and *U*_3_ vs. normalized time of the trajectories in A and B from younger larvae. (D) Same plot for older larvae. Shaded areas demarcate each cycle of the trajectory. (E–F) Postural space representation of the trajectories in C and D, respectively. Throughout, color represents the location in the behavioral space and cycle number: in the RGB colormap, red and green channels correspond to position along MDS dimension 1 and 2, respectively, and the blue channel to the cycle number.

We next used multidimensional scaling (MDS) [25] to quantify the differences between trajectories (and between the cycles of trajectories), and to determine whether they cluster into discrete behavioral patterns. Past behavioral studies have used both non-linear and linear clustering methods [19, 20], and here we applied a linear method to ensure robustness. MDS takes as input the Euclidean “distance” *d*_*αβ*_ between every pair of postural space trajectories *α* and *β* over one oscillation cycle (see Materials and Methods and S1 File), and determines the optimal low-dimensional space required to embed the distance data. In this space, shown in Fig 4A, each oscillation cycle of a trajectory is represented by a point, and the distance between two points represents how similar the trajectories in the pair are. Thus, MDS generates a “behavioral space” in which distinct behavioral patterns would appear as well-separated clusters of points.

In our analysis of an ensemble of swimming bouts, three dimensions (not to be confused with the three axes of postural space) captured ~85% of the behavioral variability contained in the set of all trajectories (S7A Fig). In Fig 4A and 4B we plot coordinate pairs 1–2 and 2–3, respectively. Points are coded as follows: filled circles are younger (7–8 dpf) and open circles older larvae (9–10 dpf). Color corresponds to position in the behavioral space and time (i.e. oscillation cycle labeled in Fig 4C and 4D): the red hue represents position along MDS dimension 1 (< 0 for less red, > 0 for more red), green hue to dimension 2 (< 0 for less green, > 0 for more green), and blue hue for cycle number (less blue for early oscillation cycles and more blue for later oscillation cycles).

The behavioral space in Fig 4A represents graphically both the temporal sequence of patterns during a swim bout and the variability in behavior among different bouts. The first oscillation cycle (points under red shaded area) captures most of this variability, spanning almost the full range of MDS dimensions 1 and 2. As seen in S7B and S7C Fig, MDS dimensions 1 and 2 correlate well with turning and scooting. Turn-like trajectories have large positive values of MDS dimension 1 and negative values of MDS dimension 2 whereas scoot-like trajectories have small values of MDS dimension 1 and positive values of MDS dimension 2. In contrast, the second oscillation cycle (points under green shaded area; see also S8A Fig) displays much less variability, with all points localized in one region. This region of behavioral space also contains the scoot-like points in cycle 1, indicating that scooting fish undergoing a scoot simply repeat the same pattern of postures over multiple cycles. Turning fish, on the other hand, generate a different oscillation cycle 1 before going to the scoot-like cycles. Further oscillation cycles (e.g. cycle 3 points under light green shaded area in Fig 4A; green, purple, blue, and gray shaded areas; see also S8A Fig) either overlap with cycle 2 or shift to a third locus of points close to the origin in behavioral space. Inspection of the data traces indicates that the latter correspond to “terminated” cycles, i.e. oscillations decaying to the resting behavior.

These observations confirm the qualitative picture formed from individual trajectories. Fig 4C and 4D color-code the amplitudes of the three eigenshapes for every recorded swim bout as in the MDS analysis in Fig 4A (trajectories from younger and older fish are plotted separately for clarity). Most of the fish-to-fish variability lies along *U*_3_ in the first cycle, as seen by inspection. Although the contribution of *U*_3_ to the zebrafish shape is less than the other two modes (<5%, see Fig 2E), it is essential to quantify fish-to-fish variation. In Fig 4E and 4F, the same set of trajectories is plotted in postural space (see also S4 and S5 Movies). As for the single trajectories in Fig 3C and S5 Fig, the postural spaces in Fig 4E and 4F show some trajectories (red) with excursions along *U*_1_–*U*_3_ (a so-called “turn region”) and others (green) lying mostly along the *U*_2_–*U*_3_ plane (a “scoot region”).

Fig 4A suggests that each cycle of a swim bout can be classified according to a region in the behavioral space. Applying a *k*-means clustering algorithm to the data in Fig 4A and using the gap method to evaluate the optimal number of clusters (see S9 Fig) reveals that behaviors can be grouped into three clusters corresponding to turn-like behavior, scoot-like behavior, and resting behavior. The first two groupings partially overlaps with classifications of scoots and R-turns based on bend angle <40° and >40° used in the literature (see S7B and S7C Fig). Nevertheless, it is important to note that the clustering is weak, with no strong boundaries in the data, and points occupying regions of behavioral space between these groupings. To assess the strength of the clustering more quantitatively, we constructed a behavioral probability distribution from the data in Fig 4A. While scoot-like behavior and rests appear as distinct peaks (S9 Fig), the turn-like behavior is not a separate maximum in the probability distribution along MDS dimensions 1 and 2, but rather a wing of the scoot-like maximum. This suggests that turn-like and scoot-like trajectories form more of a continuum of behavioral patterns than discrete behavioral states. Visual inspection of trajectories in postural space (Fig 4E and 4F) supports this view. This broad range in behavior does not appear to be a consequence of variability between fish. Trajectories from individual fish reveal the same behavioral variability within the bouts of the single fish as for the population, and there is no strong clustering of the single-fish trajectories in behavioral space (see S10A Fig).

The behavioral space also reveals differences between age groups. In Fig 4A (left panel) showing MDS dimensions 2 and 3, trajectories from younger (filled symbols) and older (open symbols) larvae occupy separate regions. The distinction with age is even more apparent for cycles 1 and 2 (S8B Fig). Inspection of Fig 4C, 4D, 4E and 4F shows that older fish display more variation in *U*_3_ in cycle 1 in comparison to younger fish; their turn interval (red trajectories) is considerably more bent than that of the younger larvae, indicating a systematic difference in shape. Nevertheless, although fish of different age seem to adopt different postures, they undergo the same temporal sequence of patterns in the behavioral space (Fig 4A).

### A simple neuro-kinematic model reproduces scoots and turns in postural space

Having established a robust, parameter-free method for analyzing zebrafish behavior, can we reproduce the observed patterns using a simple model of free swimming? We surmised that a simple neural network may suffice in describing the simple behavioral patterns observed. Constructing a network with a channel controlling the left and the right of the organism, we anticipated that signals of identical amplitude to both channels would produce a symmetric fish spine oscillation (i.e. a scoot) while unequal amplitudes would produce a turn in one direction. To test this idea, we adapted a coarse-grained neuro-kinematic model of *xenopus* swimming [23] with two input channels.

**Fig 5:**
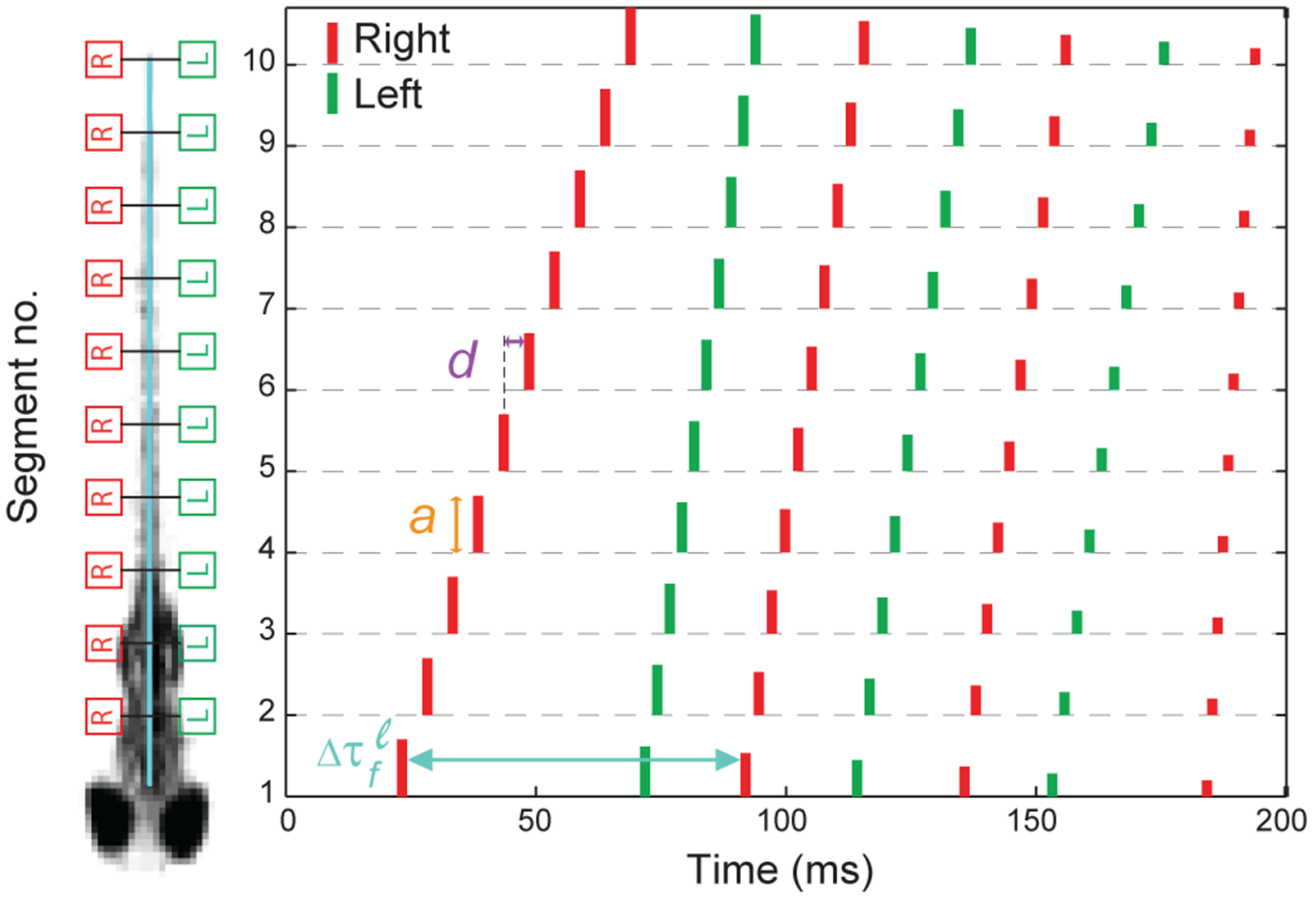
Spike train of zebrafish free swimming. (A) Schematic of neuro-kinematic model depicting the fish backbone divided into ten segments on the right and left sides. (B) Spike train generated by an optimized neural model. The right and left spike trains are shown in red and green, respectively. The height of each spike represents the amplitude *a* of the stimulus. *τ*_*f*_ is the firing time for each spike in the head segment. The segment-to-segment delay *d* is the time difference between spikes in adjacent segments of the fish backbone.

Our version of the neural network divides the fish backbone into a right and left half, each with 10 equal neural segments (*s*_*j*_) of neurons interconnected by appropriate synapses (see S11A Fig). The right and left halves each receive an input trigger signal *F*_*osc*_(*s*_*j*_, *t*_*i*_) in the form of a train of sharp pulses whose firing times *τ*_*f*_, amplitudes *a*, and segment-to-segment delays *d* are model parameters (Fig 5). The output of each neural segment along the backbone is convolved with a bi-exponential function representing the temporal response of the neuromuscular junction. The result is the force exerted by the muscles *F*_*m*_(*s*_*j*_, *t*_*i*_) at each segment on the left and right. From this force function, we determined the radius of curvature *R*(*s*_*j*_,*t*_*i*_) = *F*_*m*_(*s*_*j*_,*t*_*i*_)/*W*(*s*_*j*_) of the zebrafish backbone at each segment position, where *W*(*s*_*j*_) is the fish stiffness along its spine. Finally we calculated the tangential angles Δ*θ*(*s*_*j*_,*t*_*i*_) by integrating *R* ^-1^(*s*_*j*_,*t*_*i*_) = |*dθ*/*ds*|. The neuro-kinematic model thus produced simulated time traces of Δ*θ*(*s*_*j*_,*t*_*i*_) on which we could perform the same dimensionality reduction analysis detailed above.

**Fig 6:**
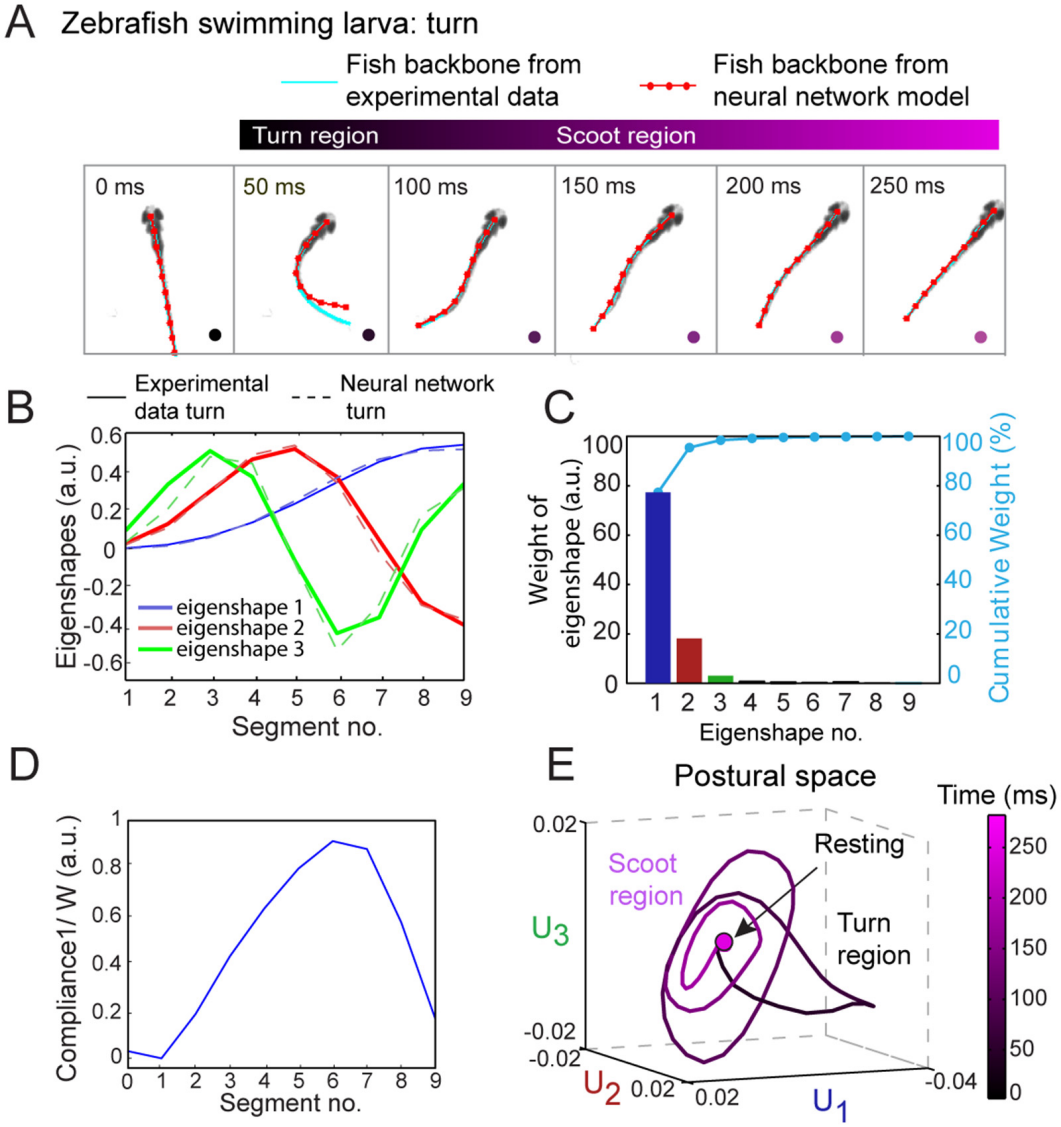
Minimal neuro-kinematic simulation of zebrafish free swimming. (A) Still images from a free swimming zebrafish movie. Each snapshot shows the fish backbone reduced to a thin skeleton fitted to a cubic spline fit (cyan) and obtained from the neural simulation (red), respectively. The neural model was optimized against the experimental data as described in S1 File. (B) Decomposition of simulated swimming traces from neuro-kinematic model into eigenshapes *V*_*k*_(*s*_*j*_). The eigenshapes from the neural model of a turn (dashed light red, blue, and green lines) match those from experimental data of a turn (solid red, blue, and green). (C) Bar plot of % weights of each eigenshapes *V*_*k*_(*s*_*j*_) (*j* = 1, 2… 9) (left axis) and cumulative contribution of each eigenshape (right axis, cyan). The first three eigenshapes contribute 98% in total to the variance in Δ*θ*. (D) Plot of head-to-tail zebrafish compliance (1/*W*(*s*_*j*_)) obtained after optimization of neuro-kinematic model against experimental data. (E) Simulated neuro-kinematic model trajectory of a turning bout in postural space.

Using a genetic algorithm, we optimized the model parameters *τ*_*f*_, *a*, and *d* and the stiffness function *W*(*s*_*j*_) against two experimental data traces (see S1 File and S11B Fig). Fig 5 shows the spike train generated by an optimized neural network model of a turn. In Fig 6A the fish backbone from neural network simulation (red dotted line) is compared with the one from experimental data (cyan). Fig 6B and 6C show that three eigenshapes are again sufficient to describe the neuro-kinematic model’s behavior. In order to simulate real larval free swimming accurately, we found that our model stiffness function *W*(*s*_*j*_) had to decrease monotonically from the head toward the tail, indicating greater flexibility towards the tail, then increase again over the last tail segment (Fig 6D). We also varied the model parameters to produce different stereotyped behaviors. We construct the postural space for the simulated fish free swimming from neural network model in Fig 6E. The simple neuro-kinematic model generates turn-like trajectories by increasing the amplitude *a* (Fig 5) to one side of the organism relative to the other and increasing the segment-to-segment delay *d* relative to a scoot during the first oscillation cycle (see S12 Fig). Thus, despite the high dimensionality of the parameter space of the model, two are sufficient to generate the observed behavioral patterns. A movie comparing a real swim and a neuro-kinematic model swim is shown in S6 Movie.

We also tested the robustness of the model by adding varying levels of white Gaussian noise to the model parameters *a*, *τ*_*f*_, and *d* (S13A and S13C Fig). We found that the free swimming behavior of the neuro-kinematic model was stable against noise as large as 70% and 90% in the amplitude *a* and delay *d*. Fluctuations in *d* simply affected the duration of the cycle. The model was most sensitive to noise in the firing time *τ*_*f*_. Not surprisingly, noise that significantly altered the timing pattern between the left and right sides of the organism affected the cyclic motion required for free swimming. A behavioral space was constructed to compare the simulated trajectories with varying signal-to-noise ratio. S13D and S13E Fig show how trajectories scattered in behavioral space with increasing noise for *a* and *τ*_*f*_, respectively, whereas the trajectories with the highest noise overlapped with those with the lowest noise in parameter *d*.

## Discussion

Prior studies of zebrafish locomotion applied boundary criteria to predefined parameters to quantify proposed behavioral states. In the current analysis, the shapes of the fish backbone themselves generate a basis set to describe their motion and reveal which basis functions contribute the most to that description. Drawing from studies of *C*. *elegans* [18] and fruit flies [19], we represent zebrafish free swimming in postural (Fig 3) and behavioral space (Fig 4). The fishes’ own motion reveal how large the behavioral space is, how free swimming trajectories are embedded in it, and how real free swimming compares to a neuro-kinematic model in behavioral space. It is worth noting that our approach sacrifices knowledge of eye angle, fin position, and other body parameters in favor of simplicity and ease of comparison to prior studies on zebrafish locomotion [9, 10].

It may be surprising at a first glance that fish larvae require only three basis functions to describe their backbone shapes, whereas worms analyzed in [18] required four. The reason is that fish are stiffer than worms thanks to their backbone (see Fig 6D), leading to a smaller repertoire of accessible shapes of the fish backbone. However, fish exhibit larger behavioral complexity. As shown in Fig 4A and 4B the behavioral space described by all collected zebrafish trajectories spans at least three dimensions. These coordinates not only capture the differences between turning and scooting bouts but also reflect unexpected differences in postures between younger and older organisms.

The behavioral space in Fig 4A helps us view swimming bouts as a sequence of postural cycles in time. During a scooting bout, the fish repeats the same cycle of postures until eventually returning to the resting behavior. This contrasts with a turning bout, during which the fish adopts a different set of backbone shapes in its first cycle, but then returns to the same postural cycles of the scooting bout. The behavioral space also reveals the level of discreteness and variability in free swimming behavior. There is a significant amount of variation in trajectories within the repertoire of the single organism, similar to that between different organisms. Although the behavioral space suggests clustering into three behavioral patterns corresponding to turns, scoots, and rests, these are not necessarily discrete, in the sense that motion between stereotyped behaviors can and does occur. In agreement with [10], where two broad overlapping peaks were found in the head angle distribution for turns and scoots, we observe a continuum of motion bounded by extremal swimming motions.

A low-dimensional postural space affords a direct visual comparison between real behavior and neural network models, as seen by comparing Figs 2C and 6E. The simple neuro-kinematic model we have adapted is robust against noise (S13 Fig) and correctly reproduces scoots as localized more in the *U*_2_–*U*_3_ plane, while turns involve *U*_1_. It also shows the bent-ellipse-like distortion of the turn interval the *U*_3_ direction, although it exaggerates it compared to real behavior. Such deviations are more obvious in behavioral space than by comparison to actual free swimming movies (S4 and S5 Movies).

A key observation from the neuro-kinematic model is that increasing the amplitude *a* of the first neural burst fed into the left/right input neurons of the model and increasing the propagation delay *d* very rapidly switches the dynamics from scoot to turn. Thus the region of behavioral space between scoots and turns is narrow in neural parameter space. Moreover, the model robustly produces free swimming patterns within the behavioral space of the real organism in the presence of noise in amplitude and segment-to-segment delay. (In contrast, the model is more sensitive to timing noise between left/right input neurons.) Of course, our simple model should not be taken to comment on the neural architecture of the real organism. For example, although our analysis suggests that turns and scoots are not distinct in terms of the cycles of postures adopted, this does not preclude them being controlled by independent neural channels in the real neuron network [26]. Rather, the model shows that a small set of parameter is sufficient to elicit all the observed swimming behaviors quantitatively. In the future, we anticipate extending these methods to the postural dynamics of escape response and prey tracking response.

## Materials and Methods

### Ethics statement

All experimental procedures in this study were approved by the University of Illinois Institutional Animal Care and Use Committee protocol # 13327.

### Animals

All experiments were performed on wild-type AB genotype zebrafish (Danio rerio) larvae age 7–10 dpf (days post fertilization). The larvae were raised without food until 6 dpf and were fed food every day (Hatchfry 0, Argent Labs) before taking data. These larvae were obtained from breeding of wild-type zebrafish adults. Zebrafish were maintained in a Z-hab mini system (Aquatic habitats, Beverly, MA) fish facility at 28.5°C on a 14h:10h light:dark cycle. The embryos were obtained from adult fish breeding and were raised at 28.5°C in 10% Hanks solution [8]. The total number of larvae used for experiments were 20: 8 (7–8 dpf) and 12 (9–10 dpf) old. All larvae were freely swimming in petri-dish before starting the experiment.

### Imaging setup

We imaged free swimming larvae using a high speed camera (IDT vision N-3) mounted to a stereo microscope (Edmund Optics 6V head + 10X eyepiece). This allowed us to image of the entire 21-mm diameter Petri dish in which the larvae swam. A halogen light source (Edmund Optics MI-150 high-intensity illuminator) was used to illuminate the sample, and its light passed through a series of long-pass filters (780 nm and 830 nm) in order to obtain IR wavelengths (>810 nm). IR light is preferable to visible light since the larva cannot detect it [9]. A circular diffuser (100 mm dia. 120 grit ground glass diffuser, Edmund Optics) was used for uniform, homogeneous illumination. The diffuser and Petri dish were mounted on a custom-built stainless steel holder on a bread board. To ensure that the movement of the larva was restricted to the x-y plane as much as possible, measurements were carried out in water 2 mm in depth.

### Behavioral experiment

For each experiment, a single 7–10 dpf fish larva was placed in a 21-mm Petri dish in 10% Hanks solution at room temperature. The larvae were illuminated from the bottom with IR light. We recorded videos of a free swimming larva at 500 fps using a high speed camera (Diagnostic Instruments) and the Motion Studio Software Suite. Each video typically had 4–5 swimming episodes ~250 ms with intermittent pauses. There were a total of 18 videos from 7–8 dpf and 21 from 8–10 dpf old fish. All videos were recorded when larvae were swimming in the center of the Petri dish, away from the dish edges that obscure the fish and making backbone shape extraction difficult. We used the frame difference method ***[27]*** with an appropriate threshold to extract swim bouts from each video. A total of 115 movies containing one free swimming bout each were collected for data analysis. All free swim movies were analyzed without selection.

### Image segmentation

All the zebrafish free swimming movies were analyzed using the image and video processing toolbox of MATLAB. A stepwise image segmentation algorithm was followed. The images were first preprocessed using a customized background subtraction algorithm. Next, the image was thresholded to 1 bit (black/white) to yield a fish outline. A skeletonization algorithm was applied to find the center of the outline, corresponding to the backbone. A cubic spline was then fitted to n points evenly sampling the fish backbone along the backbone arc length. Each step is explained in S1 File and is shown in S1 Fig.

### Eigenshape analysis

Each fish swimming bout was of 180–200 ms duration which accounts for 90–100 frames in total. We took 140 frames in total, including the resting behavior preceding and following the swimming bout of the fish. We analyzed a total of 115 swimming bouts from 20 different fish. We performed singular value decomposition as described above on the matrix Δθ(s_**j**_,t_**i**_) containing m = 16100 total frames from the catenation of all recordings of free swimming fish. To analyze multiple fish trajectories together, the time traces of the amplitudes {**U**_**k**_(**t**_**i**_)} (with k = 1, 2 and 3) were scaled and shifted in time to maximize the overlap between trajectories (S1 File). For Fig 4, we determined the “distance” between trajectories from the Euclidean distance **d**_**αβ**_ between pairs **α,β** of time-aligned spine bend angles Δ**θ** ^**α,β**^(**s**_**j**_,**t**_**i**_). Based on Eq (2) and the orthonormality of the basis functions that compose Δ**θ**, it follows that (S1 File):

$$d_{\alpha \beta }^{2}=\sum_{i}\sum_{k=1}^{n}S_{kk}^{2}{\left[U_{k}^{\alpha \;}\left({\tilde{t}}_{i}^{\alpha }\right)\;-\;U_{k}^{\beta }\left({\tilde{t}}_{i}^{\beta }\right)\right]}^{2}$$ (2)

where ${\tilde{t}}_{i}^{\alpha }$ is the normalized time for trajectory *α*.

### Neural network model

We used a modified version of the neuro-kinematic model described in [23], with 10 neurons on each side, instead of 25. A detailed description of the model is in S1 File. Briefly, the model parameters were the firing times *τ*_*f*_, stimulus amplitudes *a*, and segment-to-segment delays *d*. The number of half cycles of tail motion, *n*_c_, was based on our experimental values (6–8 half cycles observed in each swimming bout). The three model parameters were adjusted by a genetic algorithm to minimize the difference between experimental and simulated trajectories Δ*θ*(*s*_*j*_,*t*_*i*_). The genetic algorithm used 50 family members with constraints on each variable obtained from eigenshape data in each generation and typically converged after ~50 generations. The step-wise algorithm for optimization is explained in S1 File.

## Supporting Information

S1 ARRIVE Checklist NC3Rs ARRIVE Guidelines Checklist. (DOCX)

S1 Fig Segmentation of zebrafish image. (A) A user-selected region of interest (ROI) around the fish in the first frame of a movie (blue polygon) is used to create a mask. (B) Background (*B*) image obtained after interpolation of pixels at the edge of the mask into the area inside the ROI. (C) Example grayscale (*G*) image from a frame in the movie. (D) Corresponding background-subtracted image (*G*—*B*). (E) Binary image obtained from thresholding the background-subtracted image in D. (F) “Skeletonization” of the binary image. (G) Cubic spline fit to the skeletonized image in E (cyan line) with ten equally spaced points (cyan circles).
(TIF)

S2 Fig Effect of number of sampled points on zebrafish backbone decomposition into eigenshapes. Each line on the plot is the cumulative contribution of each eigenshape (as in Fig 2D) obtained for movies with different numbers of sampled points on the backbone spline: n = 3, 5, 10, 15, 20. The first three eigenshape contribute 96% of the total variance in Δ*θ* when *n* > 8. There is no significant difference in the eigenshape contribution for *n* ≥ 10.
(TIF)

S3 Fig Residual error in reconstructed spine angles using eigenshapes: The residual error between all trajectories and their reconstruction Δ*θ*(*s*_*j*_,*t*_*i*_)–Δ*θ* ^*r*^(*s*_*j*_,*t*_*i*_) using the first eigenshape (*k* = 1; top-left panel), the first two eigenshapes (*k* = 1, 2; top right), the first three eigenshapes (*k* = 1, 2, 3; bottom left) and the first four eigenshapes (*k* = 1, 2, 3, 4; bottom right). The difference in residual error is insignificant for more than 3 eigenshapes.
(TIF)

S4 Fig Real-space representation of zebrafish swimming eigenshapes. (A) Snapshots of a movie of a free swimming zebrafish recorded at 500 fps. (B) Shown are the real-space shapes (gray lines) corresponding to the basis function *V*_*k*_(*s*_*j*_) where *k* = 1, 2, 3 (left, middle, and right panels, respectively) for the swim bout shown in A. These shapes were reconstructed using only eigenshape 1, 2, 3, respectively, as described in S1 File. Each of the shapes in color corresponds to the frames marked with blue, red and green boxes in A. The zebrafish shape in the blue frame consists mostly of eigenshape *k* = 1 (left panel, solid blue line), i.e. a shape with a single bend. The zebrafish shape in the red frame consists mostly of eigenshape *k* = 2 (middle panel, solid red line), i.e. a shape with a two bends. The zebrafish shape in the green frame consists mostly of eigenshape *k* = 3 (right panel, solid green line), i.e. a shape with a three bends. The contributions from the other two eigenshape (dotted lines) for each colored frame are comparatively smaller.
(TIF)

S5 Fig Representation of scoot bout in low-dimensional postural space. (A) Still images of a representative scooting bout (from 50–250 ms) during free swimming. (B) Plot of the amplitudes *U*_1_(*t*), *U*_2_(*t*), and *U*_3_(*t*) of the three collective eigenshapes corresponding to the movie in A The regions marked by dashed lines and labeled as cycles (1–5) in *U*_1_(*t*), *U*_2_(*t*), and *U*_3_(*t*) are obtained from the oscillation cycles in *U*_1_(*t*). (C) Representation of a scoot in a postural space. The three-dimensional coordinates of the trajectory are the amplitudes *U*_1_(*t*), *U*_2_(*t*), and *U*_3_(*t*) in B. The bout entails multiple cycles (1–5) along a flat ellipse in this space. Throughout, time (0–250 ms) is represented by the black—magenta colormap.
(TIF)

S6 Fig Temporal alignment of amplitudes *U*_1_, *U*_2_, *U*_3_. (A) The amplitudes *U*_1_, *U*_2_, *U*_3_ of each eigenshape *V*_*k*_ (*k* = 1, 2, 3 in blue, red and green, respectively) for all fish swimming trajectories were aligned in time using a Lagrange multiplier optimization method. The time axes were shifted and scaled by the duration of cycle 1 to obtain maximum overlap between all sets of trajectories. Cycle 1 is the time period demarcated by the first to third zero crossings of *U*_1_, respectively (orange band). (B) Histogram of cycle 1 period for all trajectories. See S1 File for more details on the alignment.
(TIF)

S7 FigMulti-dimensional scaling of zebrafish trajectories.(A) Maximum relative error between the dissimilarity matrix $d_{\alpha \beta }^{2}$ (see Eq S8) and the MDS-reconstructed dissimilarity matrix ${d^{\prime }}_{\alpha \beta }^{2}$, plotted as a function of number of MDS dimensions. The maximum relative error was defined as $\left(d_{\alpha \beta }^{2}-{d^{\prime }}_{\alpha \beta }^{2}\right)/{d^{\prime }}_{\alpha \beta }^{2}$. Three MDS dimensions capture 85% of the variability in the trajectories. (B) Plot of fish bend angle vs. fish head angle calculated using the method described in [10] and correlation with MDS of zebrafish trajectories. Each point represents the first cycle of a trajectory. The color of each point represents the location of the trajectory in behavioral space (as in Fig 4A), with red and green corresponding to location along MDS dimension 1 and 2, respectively. Green trajectories have lower bend and head angles, corresponding to scoots, whereas red trajectories have larger bend and head angles, corresponding to turns. (C) Plot of the first cycle of trajectories in MDS dimensions 1 and 2 and correlation with fish bend angle. Trajectories classified as scoots (<40°, magenta) and turns (>40°, black) based on the fish bend angle parameters. Throughout, open circles represent older larvae (9–10 dpf), filled circles younger larvae (7–8 dpf).(TIF)

S8 Fig Behavioral space of free swimming zebrafish resolved by cycle. (A) Behavioral space in MDS dimensions 1 and 2 from Fig 4A, plotting each oscillation cycle separately (leftmost panel for cycle 1, rightmost panel for cycle 6). (B) Behavioral space in MDS dimensions 2 and 3 from Fig 4B, plotting each oscillation cycle separately. Throughout, the same colormap and symbols from Fig 4 are used.
(TIF)

S9 Fig Clustering analysis of zebrafish free swimming behavior. (A) *K*-means clustering of the trajectories in Fig 4. The optimal number of clusters were evaluated using the “gap” method [28], which searches for gaps in the data. The gap value criterion reaches a maximum at three, showing that the data are best described by three clusters. (B) Plot of behavioral space data from Fig 4A clustered into three groups, corresponding roughly to turns (red circles), scoots (green diamonds), and rests (blue x’s). (C) Plot of behavioral space probability distribution. Density kernel estimation [29]was used to construct the distribution from the data points (Gaussian widths of σ_1_ = 275 and σ_2_ = 80 along MDS dimensions 1 and 2 were used, respectively).
(TIF)

S10 Fig Behavioral variability in a single zebrafish vs. population. (A) Behavioral space in MDS dimensions 1 and 2 from Fig 4A with each fish (*N* = 20) represented by a separate color (see colormap). Older larvae are represented by open circles, younger by filled circles. The same level of behavioral variability is observed at the single-fish level as at the population level. Individual fish do not exhibit any preference for one type of behavior pattern. (B) Behavioral space in MDS dimensions 2 and 3 from Fig 4B with each fish represented different colors as in A. Fish trajectories separate by age as demarcated by the elliptical outlines (solid for younger, dashed for older larvae).
(TIF)

S11 Fig Neural Model. (A) A schematic of a neural model depicting the fish backbone divided into ten segments on either side of the backbone. (B-C) Examples of Δ*θ*(*s*_*j*_,*t*_*i*_) for a turn and a scoot trajectory, respectively. The zero crossings of Δ*θ*(*s*_*j*_,*t*_*i*_) are labeled at *j* = 1 (close to the head), 9 (tail) in dotted and solid lines, respectively. These are used to calculate the segment-to-segment delay of the neural signal as described in S1 File.
(TIF)

S12 FigSpike train of a scooting bout.(A) Schematic of neuro-kinematic model depicting the fish backbone divided into ten segments on the right and left sides. (B) Spike train generated by an optimized neural model. The right and left spike trains are shown in red and green, respectively. The height of each spike represents the amplitude *a* of the stimulus. $\Delta \tau_{f}^{l}$ is the firing time difference for right side half cycles. The segment-to-segment delay *d* is the time difference between spikes in adjacent segments of the fish backbone.(TIF)

S13 FigEffect of noise on neural model.(A-C) Response of the neural model to white Gaussian noise added to the model parameters $\left\{a_{l},\tau_{f}^{l},d_{l}\right\}$, respectively, for models that produce scoots (green) and turns (red). As described in S1 File, the response is calculated as $d_{\alpha \beta (noise)}^{2}-d_{\alpha \beta (neuro)}^{2}$. (D-F) The first cycle of the simulated trajectories with noise added to the parameters $\left\{a_{l},\tau_{f}^{l},d_{l}\right\}$, respectively, embedded in the same behavioral space as Fig 4A. Simulated trajectories with signal-to-noise ratio (snr) = 1, 6, and ∞ are shown in triangles, squares, and diamonds, respectively. Red and green colored symbols of each kind represent simulated turning and scooting trajectories, respectively.(TIF)

S1 File Methods. Contains additional information on data analysis methods, and details on the neural network model.
(DOCX)

S1 Movie Free swimming zebrafish larva. Video of a freely swimming larva in a petri dish recorded at a rate of 500 frames per second (fps) using a high-speed camera. The video shows a total of 3 swimming episodes with 4 intermittent pauses (resting). The playback rate is 100 fps.
(MP4)

S2 Movie Postural space representation of zebrafish larva turning bout. The left side of the movie shows free swimming larva at 10 fps and a spline curve in cyan fitted to its backbone. The right side shows the corresponding trajectory in postural space. The dot shows the contribution of amplitudes *U*_1_(*t*), *U*_2_(*t*), and *U*_3_(*t*) of eigenfunction *V*_1_, *V*_2_, and *V*_3_, respectively, for the corresponding frame on the left. In postural space, the bout involves a turn region (*t* = 50–100 ms), represented as a bent ellipse, followed by a scoot region (*t* = 100–250 ms) represented as multiple cycles along flat ellipses, and a final return to the rest behavior. Throughout, time (0–250 ms) is represented by the black—magenta colormap.
(MP4)

S3 Movie Postural space representation of a zebrafish larva scoot. The left side of the movie shows a free swimming larva (at 10fps) and a spline curve in cyan fitted to its backbone. The right side of the movie shows the corresponding trajectory in postural space. The dot shows the contribution of amplitudes *U*_1_(*t*), *U*_2_(*t*), and *U*_3_(*t*) of eigenfunction *V*_1_, *V*_2_, and *V*_3_, respectively, for the corresponding frame on the left. In postural space, the bout involves a scoot region (*t* = 50–250 ms) represented as multiple cycles along flat ellipses, and a final return to the rest behavior. Throughout, time (0–250 ms) is represented by the black—magenta colormap.
(MP4)

S4 Movie Postural space of a population of younger zebrafish (7–8 dpf). The movie shows the trajectories from a population of younger fish in postural space. The animation sweeps over elevation angle to show how trajectories occupy a continuum between two extremes—turn-like trajectories and scoot-like trajectories—rather than clustering into distinct behavioral states. For details on the colormap see Fig 4.
(MP4)

S5 Movie Postural space of a population of older zebrafish (9–10 dpf). The movie shows the trajectories from a population of older fish in postural space. The animation sweeps over elevation angle to show how trajectories occupy a continuum between two extremes—turn-like trajectories and scoot-like trajectories—rather than clustering into distinct behavioral states. For details on the colormap see Fig 4.
(MP4)

S6 Movie Simulated turn and scoot of zebrafish using neural network model. The left side of the movie shows a free swimming larva recorded at a rate of 500 fps and a spline curve in cyan fitted to its backbone. The right side shows the same free swimming larva and a spline curve obtained using the neural network model described in the text. The movie has two swimming episodes (a turning bout followed by a scooting bout) with one intermittent pause. The colored marker represents the tail position at each time point. The playback rate is 10 fps.
(MP4)
