# Supplementary material for: The Behavioral Space of Zebrafish Locomotion and Its Neural Network Analog
Source: PLoS One. 2015 Jul 1;10(7):e0128668. doi: 10.1371/journal.pone.0128668 (PMC4489106; doi:10.1371/journal.pone.0128668)

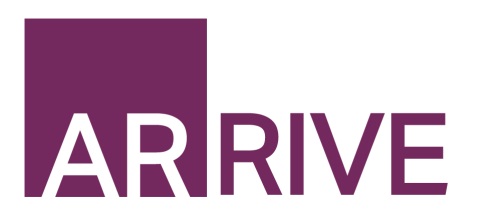


The ARRIVE Guidelines Checklist

Animal Research: Reporting In Vivo Experiments

Carol Kilkenny^1^, William J Browne^2^, Innes C Cuthill^3^, Michael Emerson^4^ and Douglas G Altman^5^

*^1^The National Centre for the Replacement, Refinement and Reduction of Animals in Research, London, UK, ^2^School of Veterinary Science, University of Bristol, Bristol, UK, ^3^School of Biological Sciences, University of Bristol, Bristol, UK, ^4^National Heart and Lung Institute, Imperial College London, UK, ^5^Centre for Statistics in Medicine, University of Oxford, Oxford, UK.*

|  | | ITEM | RECOMMENDATION | Section/ Paragraph |
| --- | --- | --- | --- | --- |
| 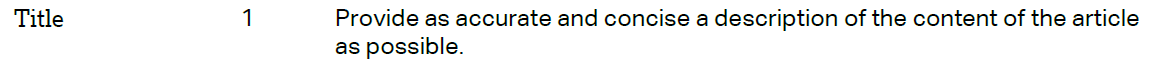 | | | Title |  |
| 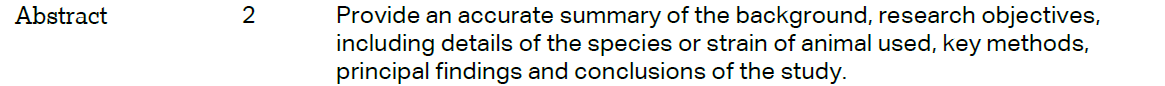 | | | Abstract |  |
| INTRODUCTION | | |  |  |
| 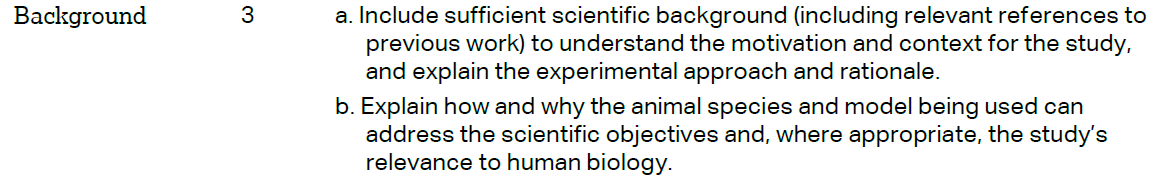 | | | Paragraph 1-4  Paragraph 5 |  |
| 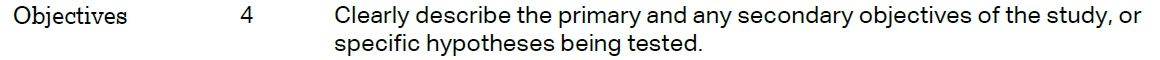 | | | Paragraph 4-5 |  |
| METHODS | | |  |  |
| 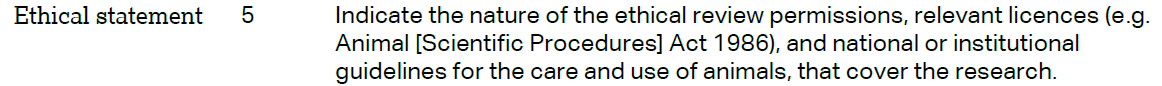 | | | Paragraph 1 |  |
| 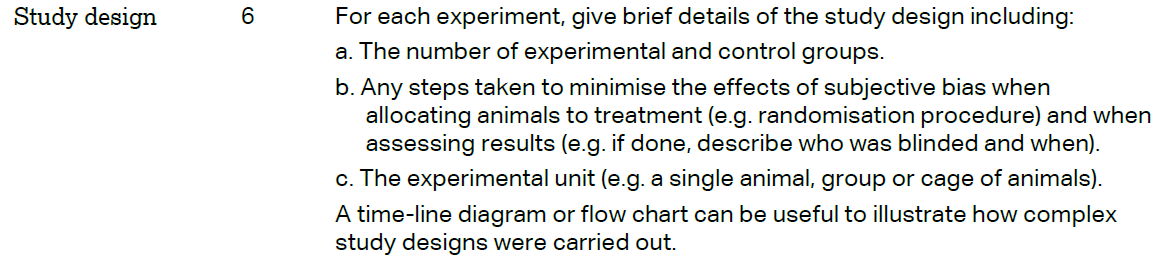 | | | Paragraph 4  N/A  Paragraph 4 |  |
| 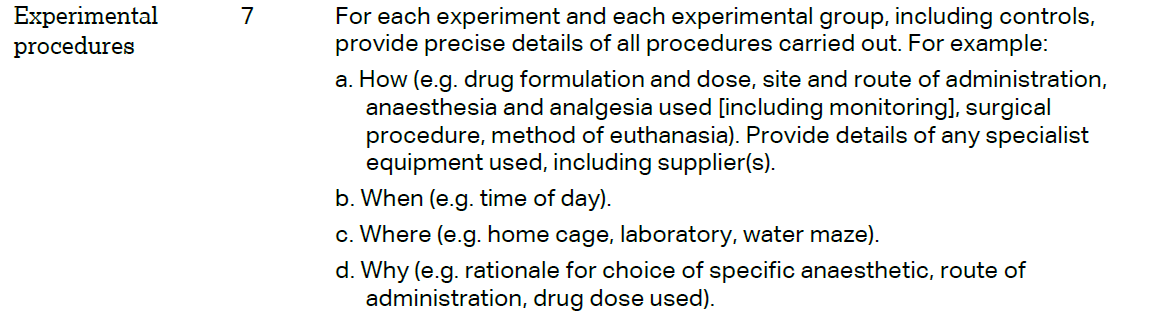 | | | Paragraph 2  Paragraph 2  Paragraph 2  Paragraph 2 |  |
| 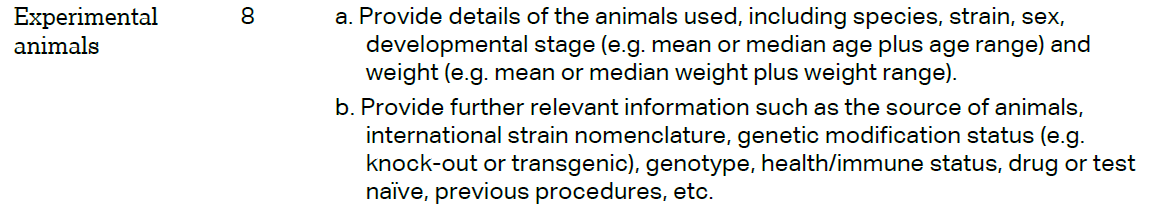 | | | Paragraph 2  Paragraph 2 |  |

The ARRIVE guidelines. Originally published in *PLoS Biology*, June 2010^1^

| 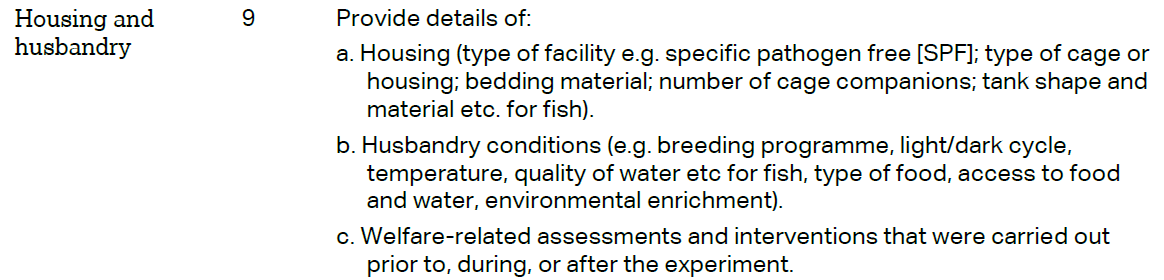 | Paragraph 2  Paragraph 2  Paragraph 2 | |
| --- | --- | --- |
| 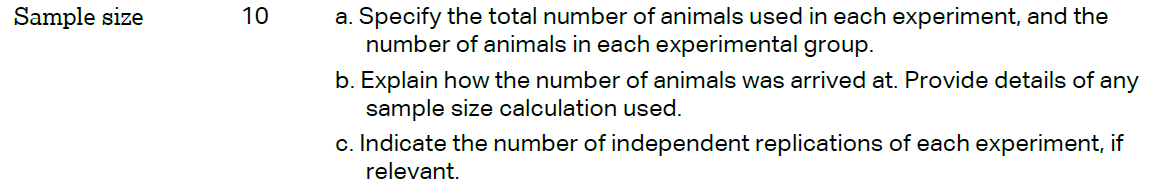 | Paragraph 1, 4  N/A  N/A | |
| 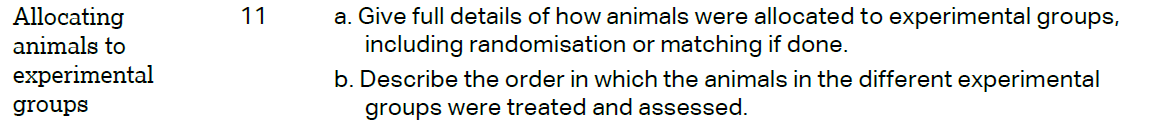 | N/A  N/A | |
| 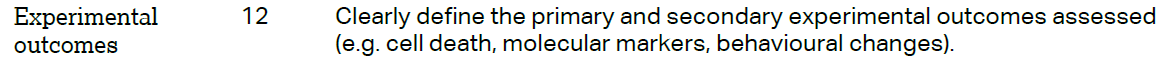 | Throughout the results section | |
| 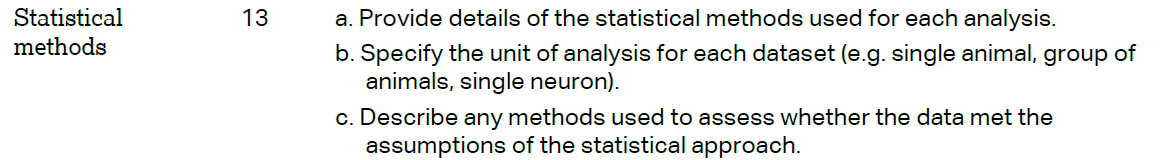 | Paragraph 6  Paragraph 4  Results/Paragraph 8 | |
| RESULTS |  | |
| 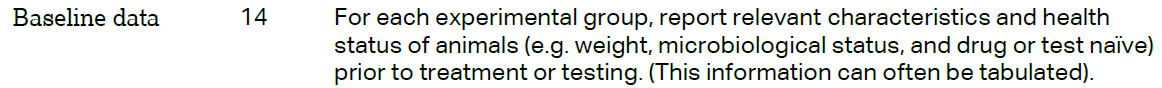 | Materials and Methods/  Paragraph 2 | |
| 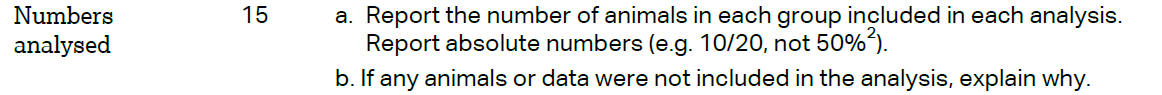 | Figure 4  N/A | |
| 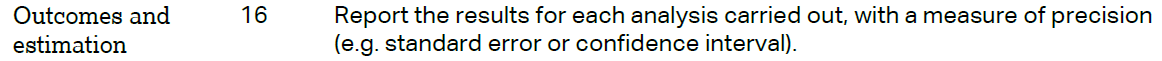 | Figure 2 and Figure S7A | |
| 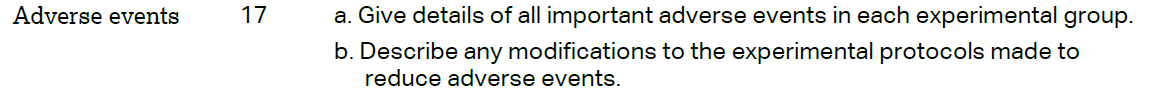 | N/A  N/A | |
| DISCUSSION |  | |
| 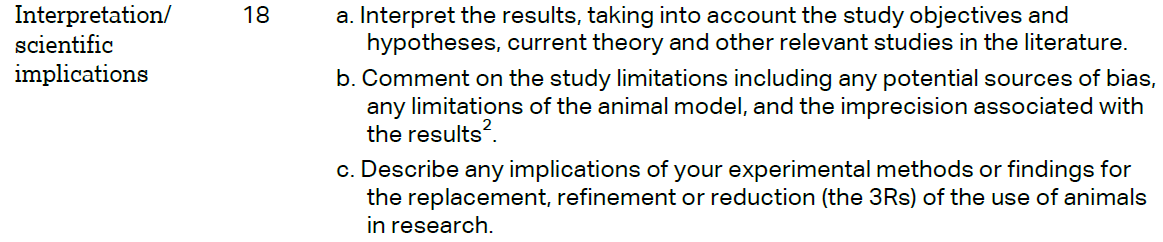 | Throughout  Paragraph 5  N/A | |
| 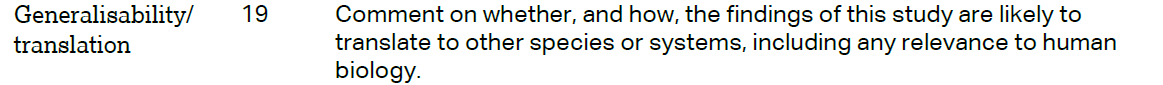 | N/A | |
| 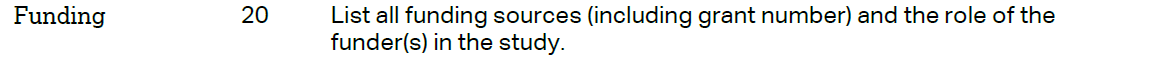 | |  |


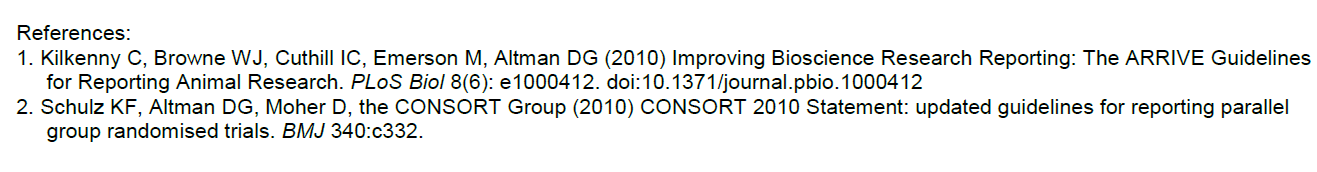

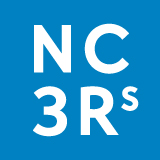

Supplement: S1 ARRIVE Checklist — (DOCX) [file pone.0128668.s001.docx]
