# Supplementary figures and images for: The Behavioral Space of Zebrafish Locomotion and Its Neural Network Analog
Source: PLoS One. 2015 Jul 1;10(7):e0128668. doi: 10.1371/journal.pone.0128668 (PMC4489106; doi:10.1371/journal.pone.0128668)

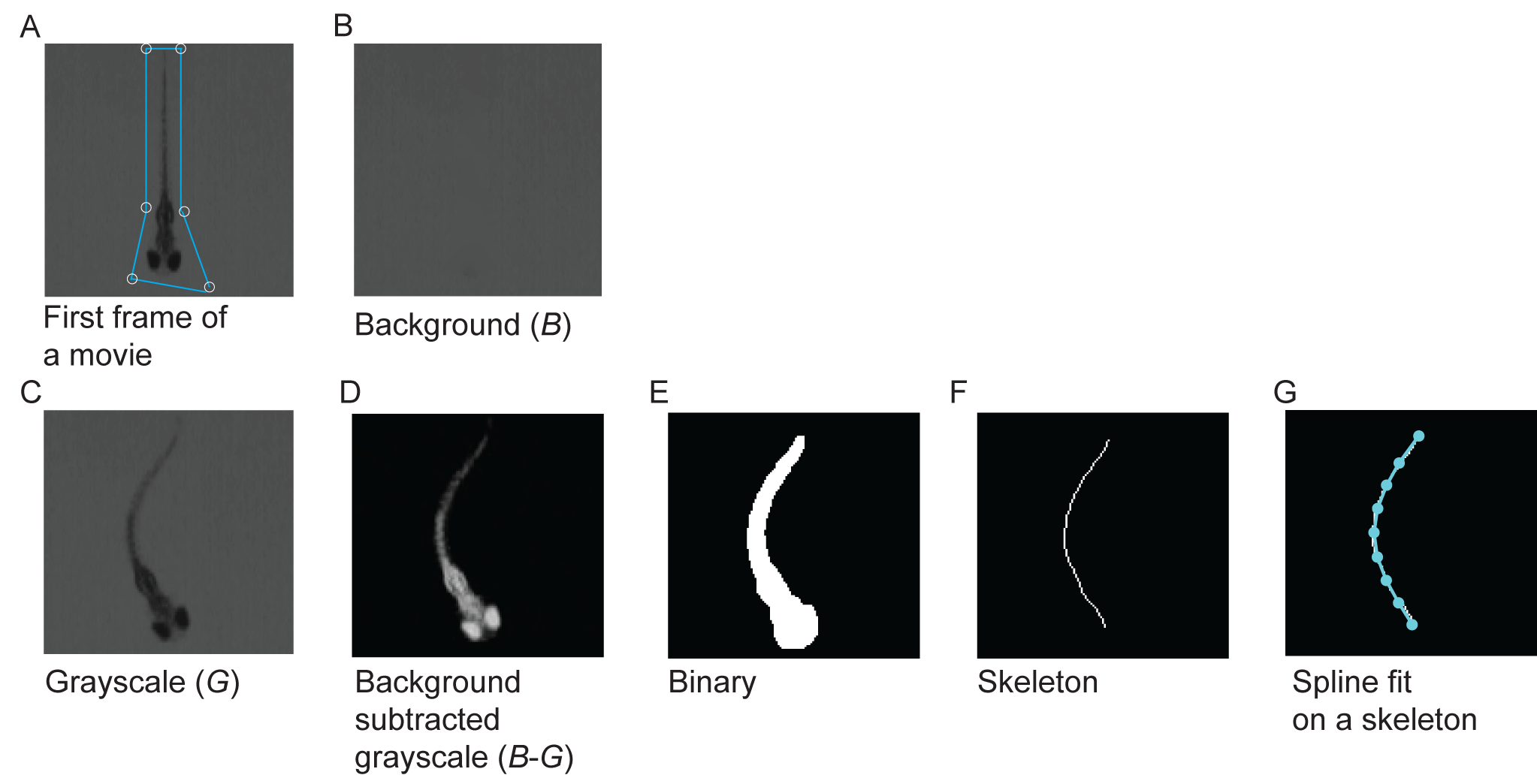

Supplement: S1 Fig — (A) A user-selected region of interest (ROI) around the fish in the first frame of a movie (blue polygon) is used to create a mask. (B) Background (B) image obtained after interpolation of pixels at the edge of the mask into the area inside the ROI. (C) Example grayscale (G) image from a frame in the movie. (D) Corresponding background-subtracted image (G—B). (E) Binary image obtained from thresholding the background-subtracted image in D. (F) “Skeletonization” of the binary image. (G) Cubic spline fit to the skeletonized image in E (cyan line) with ten equally spaced points (cyan circles). (TIF) [file pone.0128668.s002.tif]

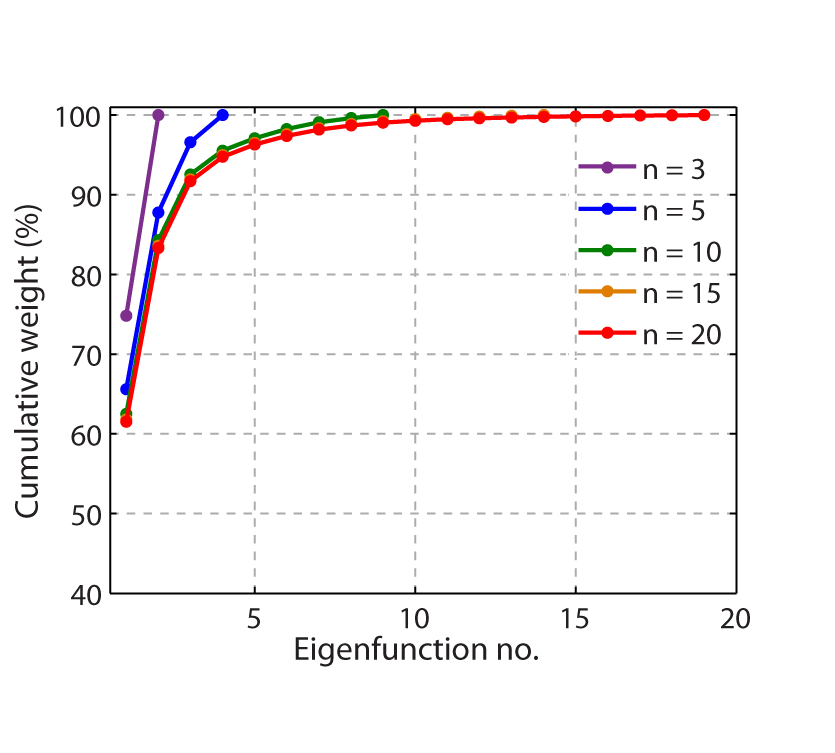

Supplement: S2 Fig — Each line on the plot is the cumulative contribution of each eigenshape (as in Fig 2D) obtained for movies with different numbers of sampled points on the backbone spline: n = 3, 5, 10, 15, 20. The first three eigenshape contribute 96% of the total variance in Δθ when n > 8. There is no significant difference in the eigenshape contribution for n ≥ 10. (TIF) [file pone.0128668.s003.tif]

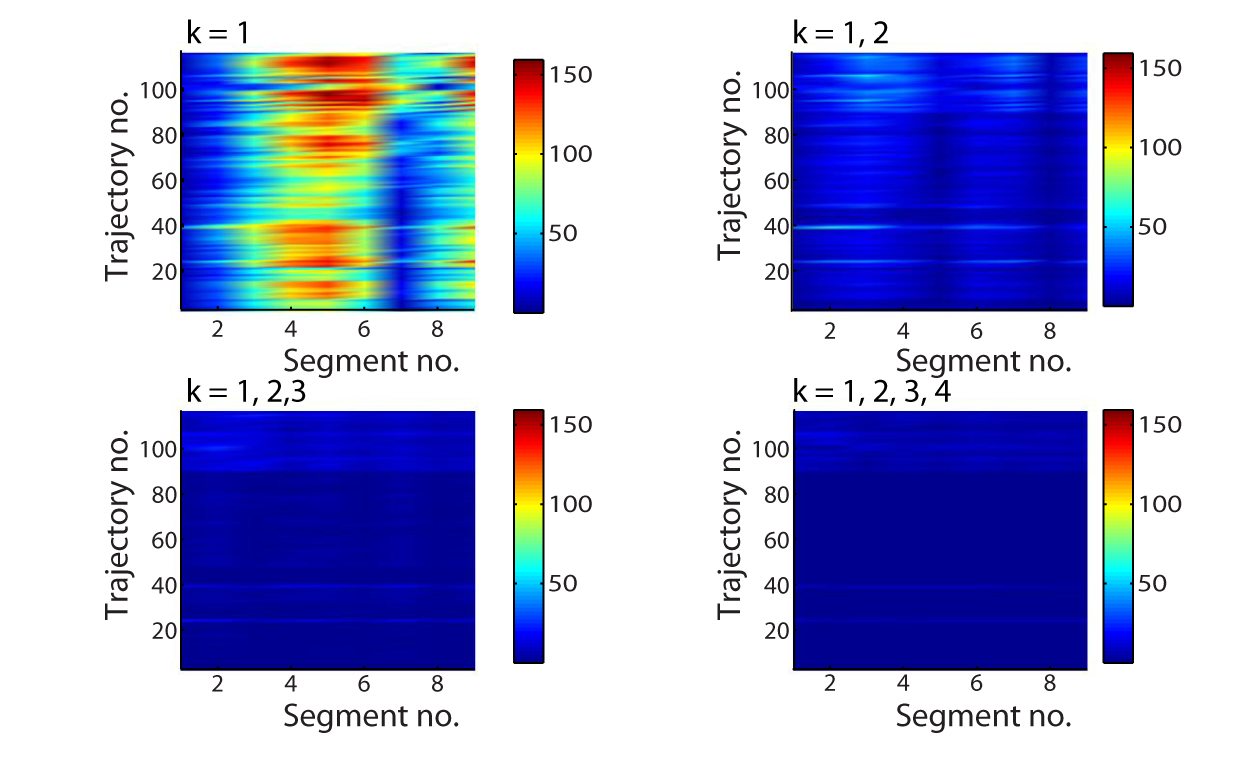

Supplement: S3 Fig — The difference in residual error is insignificant for more than 3 eigenshapes. (TIF) [file pone.0128668.s004.tif]

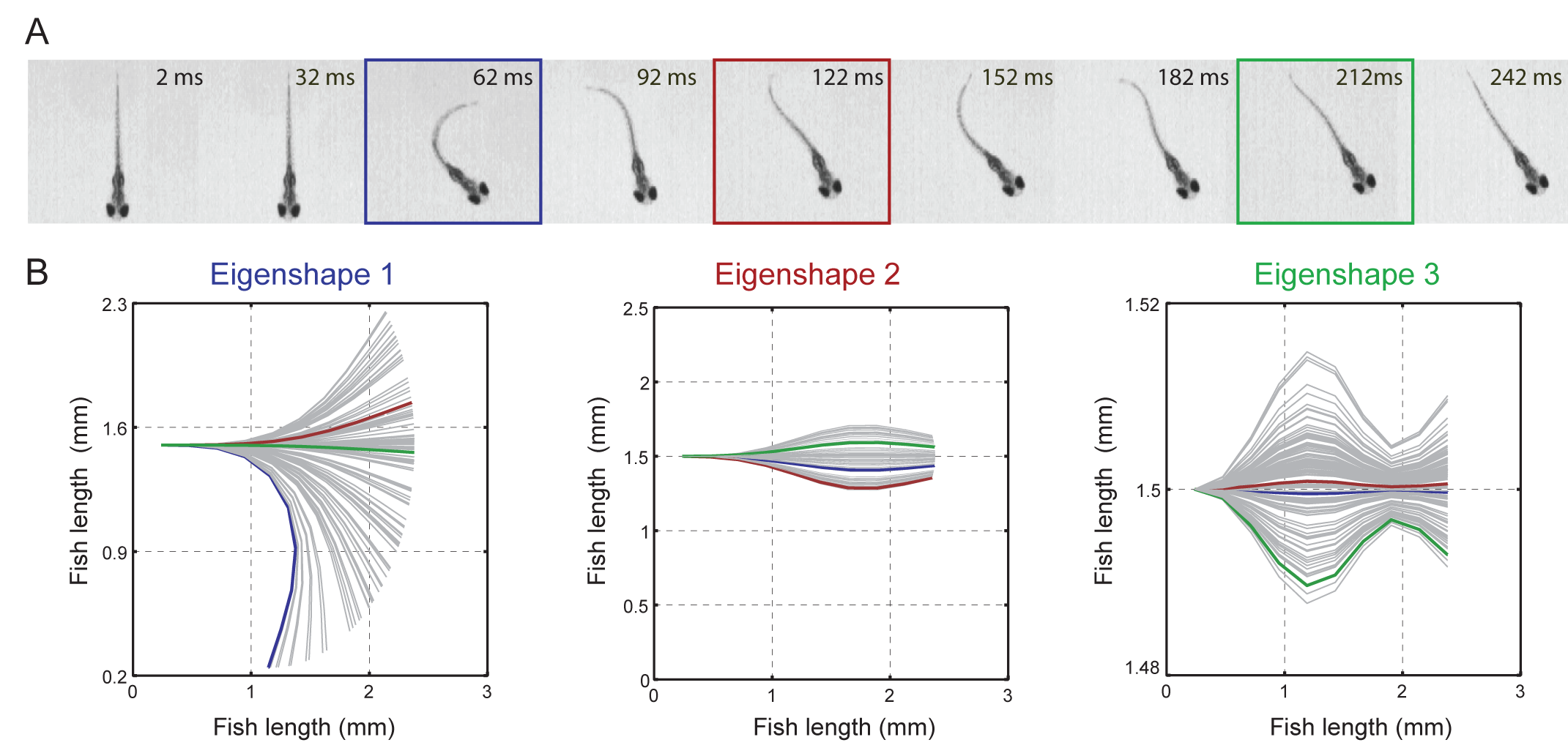

Supplement: S4 Fig — (A) Snapshots of a movie of a free swimming zebrafish recorded at 500 fps. (B) Shown are the real-space shapes (gray lines) corresponding to the basis function V k(s j) where k = 1, 2, 3 (left, middle, and right panels, respectively) for the swim bout shown in A. These shapes were reconstructed using only eigenshape 1, 2, 3, respectively, as described in S1 File. Each of the shapes in color corresponds to the frames marked with blue, red and green boxes in A. The zebrafish shape in the blue frame consists mostly of eigenshape k = 1 (left panel, solid blue line), i.e. a shape with a single bend. The zebrafish shape in the red frame consists mostly of eigenshape k = 2 (middle panel, solid red line), i.e. a shape with a two bends. The zebrafish shape in the green frame consists mostly of eigenshape k = 3 (right panel, solid green line), i.e. a shape with a three bends. The contributions from the other two eigenshape (dotted lines) for each colored frame are comparatively smaller. (TIF) [file pone.0128668.s005.tif]

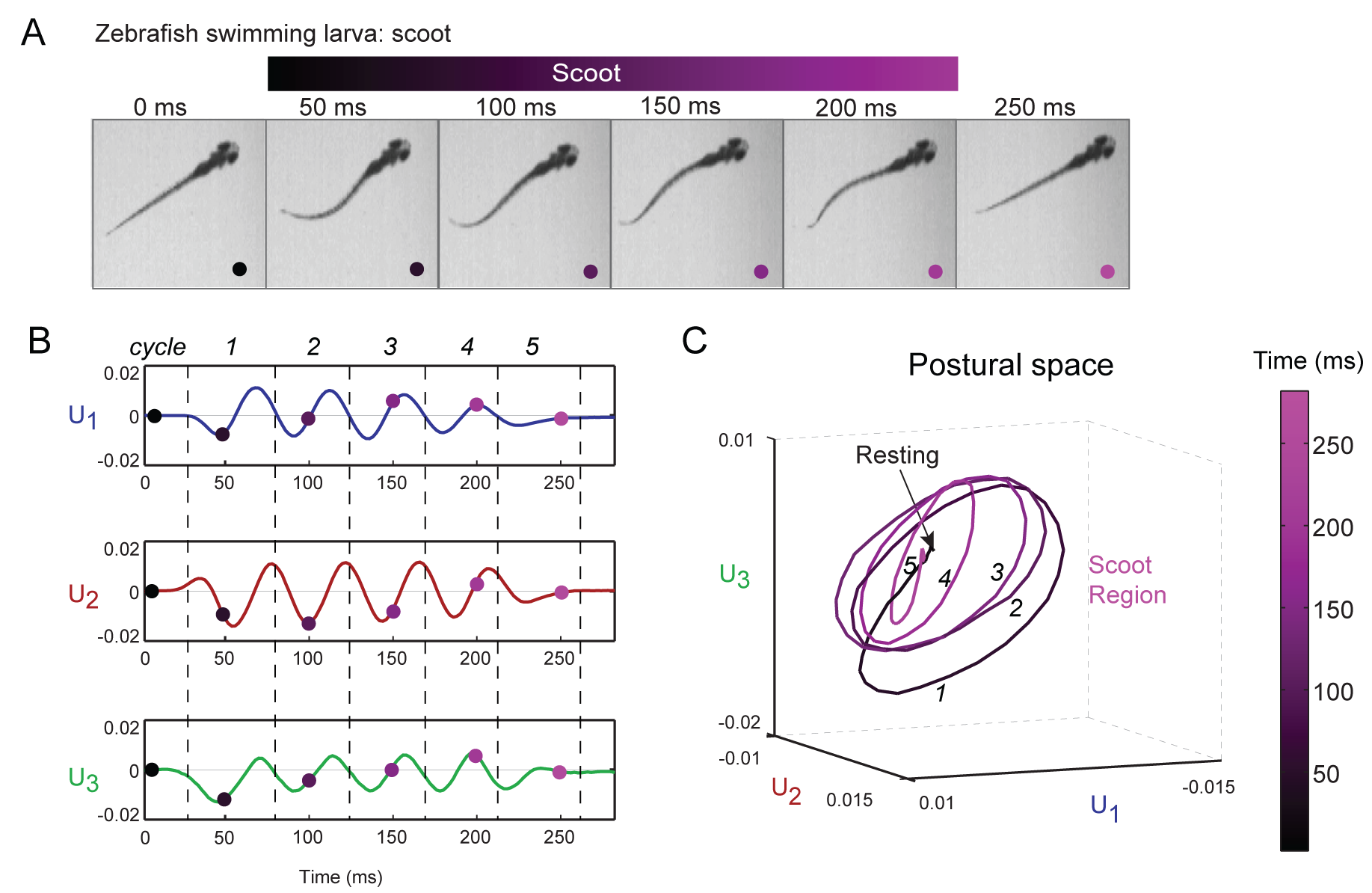

Supplement: S5 Fig — (A) Still images of a representative scooting bout (from 50–250 ms) during free swimming. (B) Plot of the amplitudes U 1(t), U 2(t), and U 3(t) of the three collective eigenshapes corresponding to the movie in A The regions marked by dashed lines and labeled as cycles (1–5) in U 1(t), U 2(t), and U 3(t) are obtained from the oscillation cycles in U 1(t). (C) Representation of a scoot in a postural space. The three-dimensional coordinates of the trajectory are the amplitudes U 1(t), U 2(t), and U 3(t) in B. The bout entails multiple cycles (1–5) along a flat ellipse in this space. Throughout, time (0–250 ms) is represented by the black—magenta colormap. (TIF) [file pone.0128668.s006.tif]

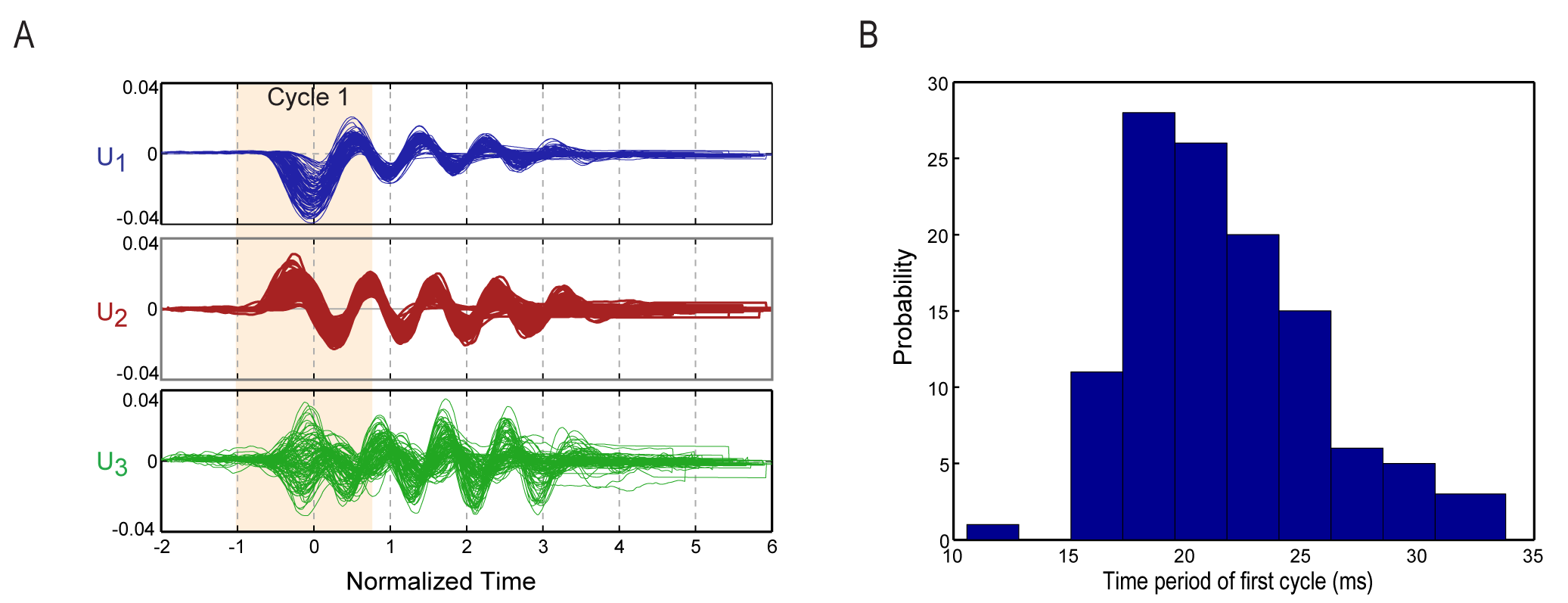

Supplement: S6 Fig — (A) The amplitudes U 1, U 2, U 3 of each eigenshape V k (k = 1, 2, 3 in blue, red and green, respectively) for all fish swimming trajectories were aligned in time using a Lagrange multiplier optimization method. The time axes were shifted and scaled by the duration of cycle 1 to obtain maximum overlap between all sets of trajectories. Cycle 1 is the time period demarcated by the first to third zero crossings of U 1, respectively (orange band). (B) Histogram of cycle 1 period for all trajectories. See S1 File for more details on the alignment. (TIF) [file pone.0128668.s007.tif]

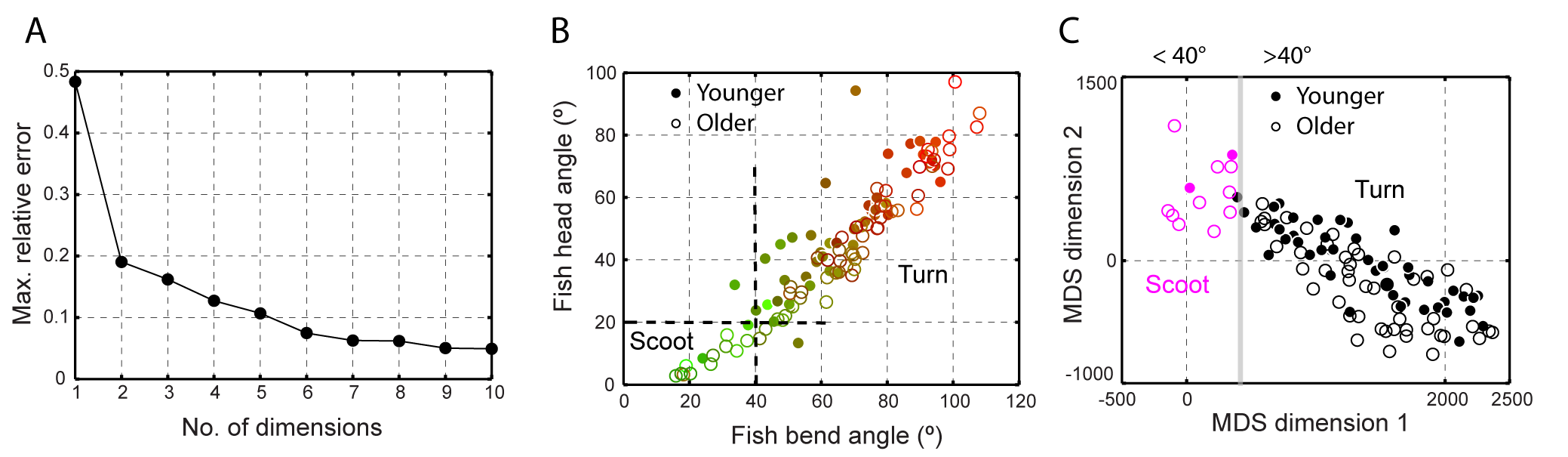

Supplement: S7 Fig — (A) Maximum relative error between the dissimilarity matrix dαβ2 (see Eq S8) and the MDS-reconstructed dissimilarity matrix d′αβ2, plotted as a function of number of MDS dimensions. The maximum relative error was defined as (dαβ2−d′αβ2)/d′αβ2. Three MDS dimensions capture 85% of the variability in the trajectories. (B) Plot of fish bend angle vs. fish head angle calculated using the method described in [10] and correlation with MDS of zebrafish trajectories. Each point represents the first cycle of a trajectory. The color of each point represents the location of the trajectory in behavioral space (as in Fig 4A), with red and green corresponding to location along MDS dimension 1 and 2, respectively. Green trajectories have lower bend and head angles, corresponding to scoots, whereas red trajectories have larger bend and head angles, corresponding to turns. (C) Plot of the first cycle of trajectories in MDS dimensions 1 and 2 and correlation with fish bend angle. Trajectories classified as scoots (<40°, magenta) and turns (>40°, black) based on the fish bend angle parameters. Throughout, open circles represent older larvae (9–10 dpf), filled circles younger larvae (7–8 dpf). (TIF) [file pone.0128668.s008.tif]

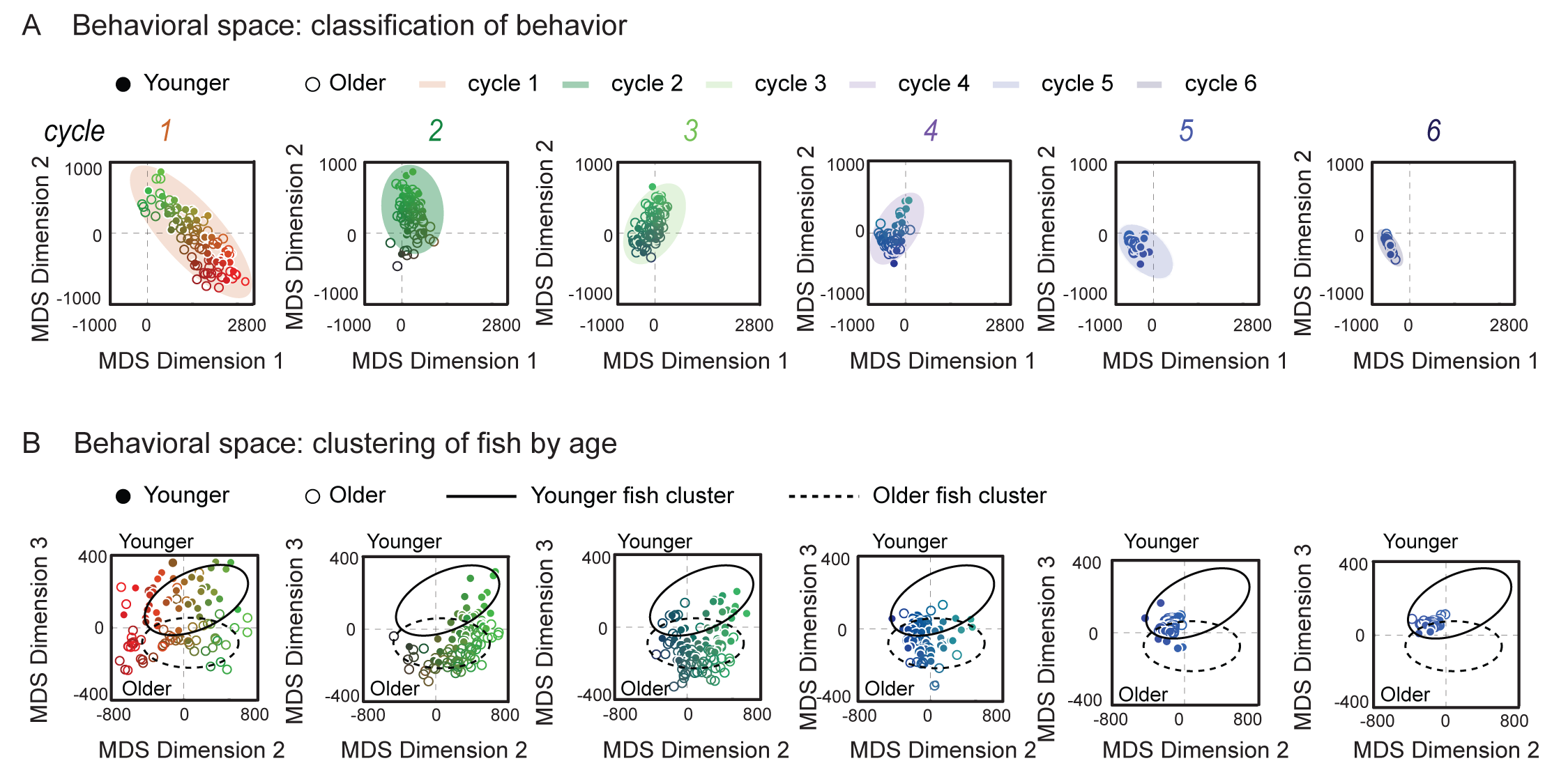

Supplement: S8 Fig — (A) Behavioral space in MDS dimensions 1 and 2 from Fig 4A, plotting each oscillation cycle separately (leftmost panel for cycle 1, rightmost panel for cycle 6). (B) Behavioral space in MDS dimensions 2 and 3 from Fig 4B, plotting each oscillation cycle separately. Throughout, the same colormap and symbols from Fig 4 are used. (TIF) [file pone.0128668.s009.tif]

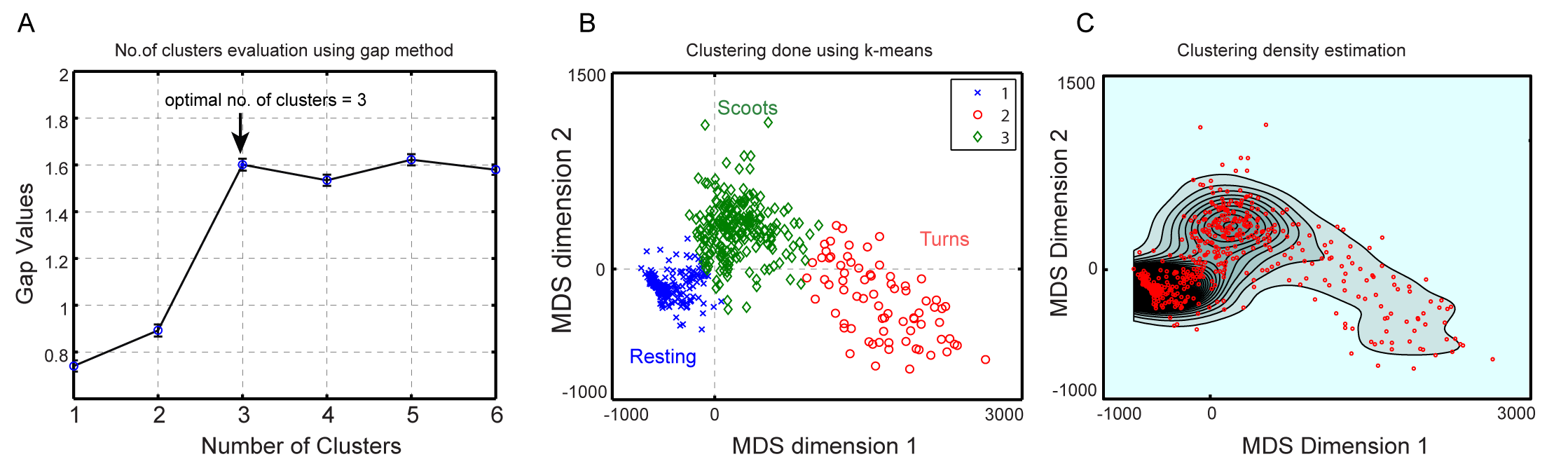

Supplement: S9 Fig — (A) K-means clustering of the trajectories in Fig 4. The optimal number of clusters were evaluated using the “gap” method [28], which searches for gaps in the data. The gap value criterion reaches a maximum at three, showing that the data are best described by three clusters. (B) Plot of behavioral space data from Fig 4A clustered into three groups, corresponding roughly to turns (red circles), scoots (green diamonds), and rests (blue x’s). (C) Plot of behavioral space probability distribution. Density kernel estimation [29]was used to construct the distribution from the data points (Gaussian widths of σ1 = 275 and σ2 = 80 along MDS dimensions 1 and 2 were used, respectively). (TIF) [file pone.0128668.s010.tif]

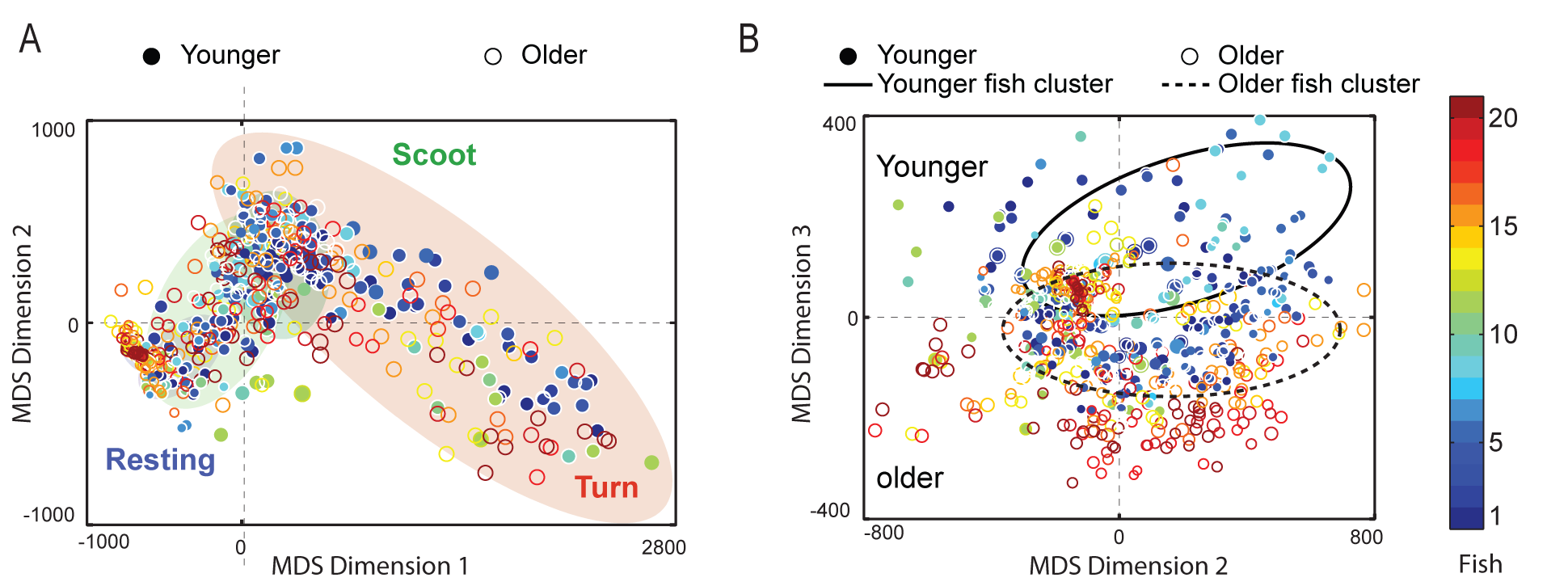

Supplement: S10 Fig — (A) Behavioral space in MDS dimensions 1 and 2 from Fig 4A with each fish (N = 20) represented by a separate color (see colormap). Older larvae are represented by open circles, younger by filled circles. The same level of behavioral variability is observed at the single-fish level as at the population level. Individual fish do not exhibit any preference for one type of behavior pattern. (B) Behavioral space in MDS dimensions 2 and 3 from Fig 4B with each fish represented different colors as in A. Fish trajectories separate by age as demarcated by the elliptical outlines (solid for younger, dashed for older larvae). (TIF) [file pone.0128668.s011.tif]

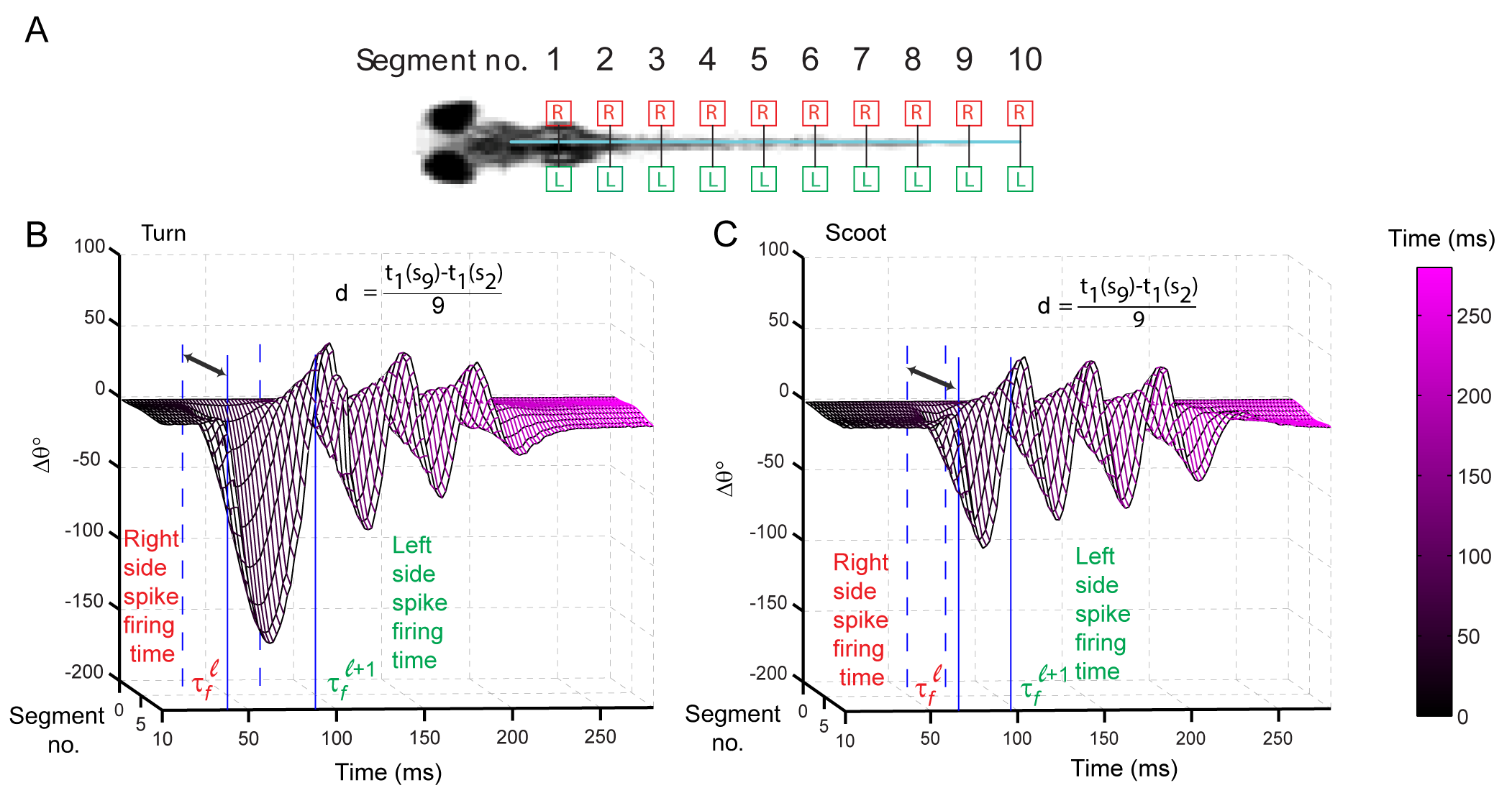

Supplement: S11 Fig — (A) A schematic of a neural model depicting the fish backbone divided into ten segments on either side of the backbone. (B-C) Examples of Δθ(s j,t i) for a turn and a scoot trajectory, respectively. The zero crossings of Δθ(s j,t i) are labeled at j = 1 (close to the head), 9 (tail) in dotted and solid lines, respectively. These are used to calculate the segment-to-segment delay of the neural signal as described in S1 File. (TIF) [file pone.0128668.s012.tif]

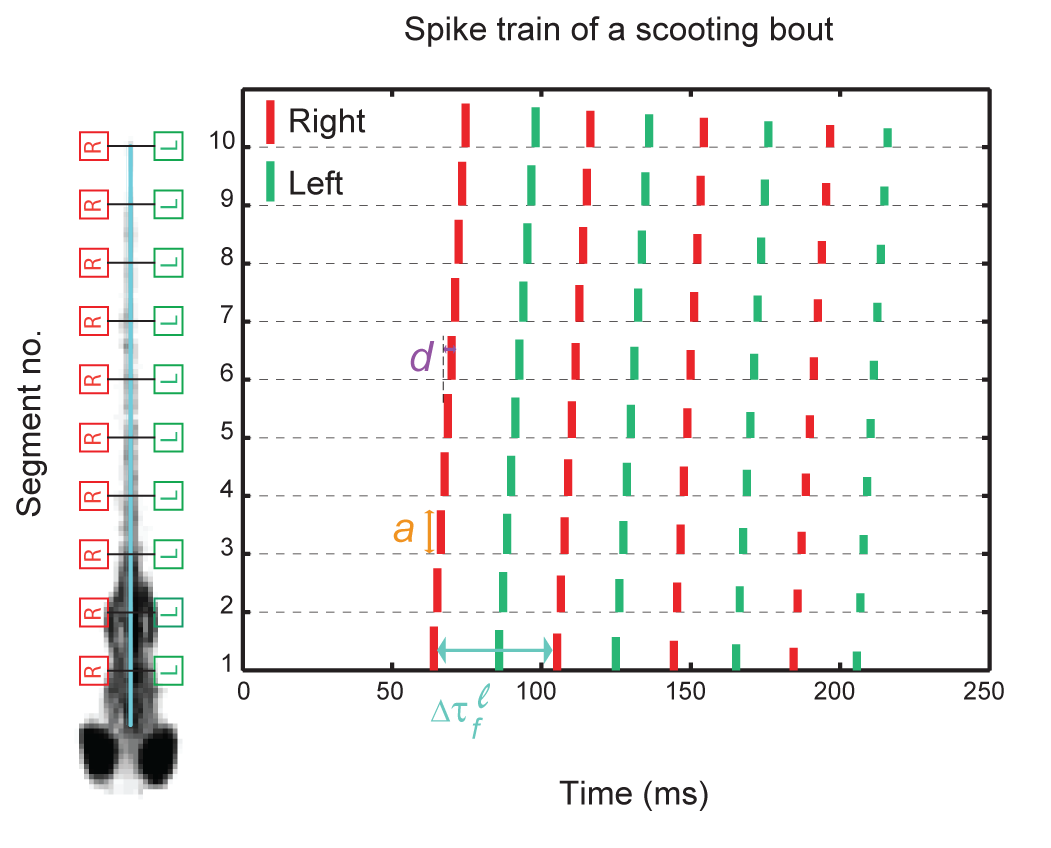

Supplement: S12 Fig — (A) Schematic of neuro-kinematic model depicting the fish backbone divided into ten segments on the right and left sides. (B) Spike train generated by an optimized neural model. The right and left spike trains are shown in red and green, respectively. The height of each spike represents the amplitude a of the stimulus. Δτfl is the firing time difference for right side half cycles. The segment-to-segment delay d is the time difference between spikes in adjacent segments of the fish backbone. (TIF) [file pone.0128668.s013.tif]

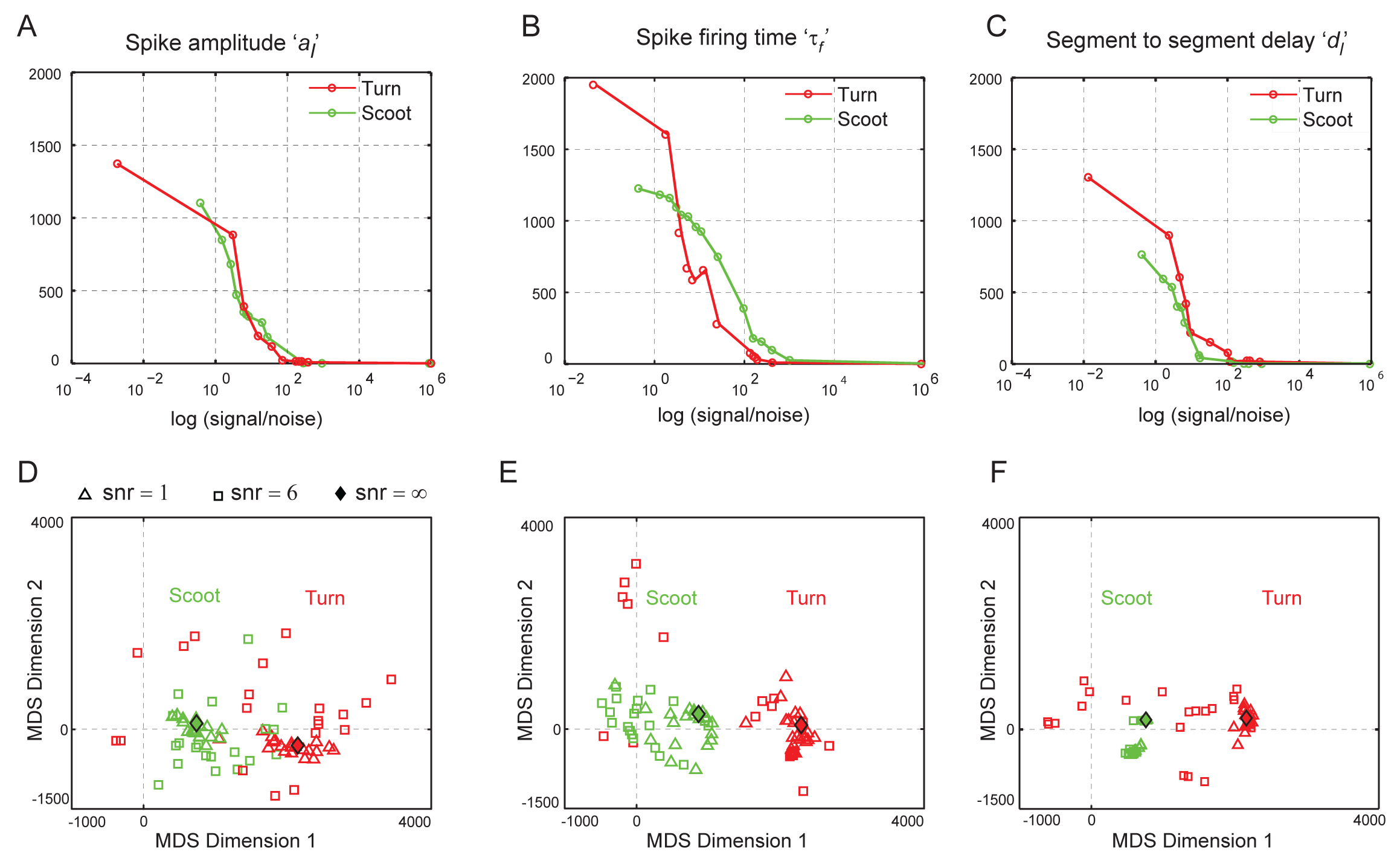

Supplement: S13 Fig — (A-C) Response of the neural model to white Gaussian noise added to the model parameters {al,τfl,dl}, respectively, for models that produce scoots (green) and turns (red). As described in S1 File, the response is calculated as dαβ(noise)2−dαβ(neuro)2. (D-F) The first cycle of the simulated trajectories with noise added to the parameters {al,τfl,dl}, respectively, embedded in the same behavioral space as Fig 4A. Simulated trajectories with signal-to-noise ratio (snr) = 1, 6, and ∞ are shown in triangles, squares, and diamonds, respectively. Red and green colored symbols of each kind represent simulated turning and scooting trajectories, respectively. (TIF) [file pone.0128668.s014.tif]
