# Supplementary material for: The Behavioral Space of Zebrafish Locomotion and Its Neural Network Analog
Source: PLoS One. 2015 Jul 1;10(7):e0128668. doi: 10.1371/journal.pone.0128668 (PMC4489106; doi:10.1371/journal.pone.0128668)
Supplement: S1 File — Contains additional information on data analysis methods, and details on the neural network model. (DOCX) [file pone.0128668.s015.docx]

**Supporting Information**

**The behavioral space of zebrafish locomotion and its neural network analog**

**Kiran Girdhar,^1^ Martin Gruebele,^1,2,3*^ Yann R. Chemla,^1,2*^**

^1^Center for Biophysics and Computational Biology, University of Illinois, Urbana IL 61801, USA;

^2^Department of Physics, Center for the Physics of Living Cells, University of Illinois, Urbana IL 61801 USA;

^3^Department of Chemistry, University of Illinois, Urbana 61801, USA;

**Methods**

**Image segmentation**

All zebrafish swimming movies were analyzed using the image and video processing toolbox of MATLAB. A stepwise image segmentation algorithm was followed. All recorded movies were converted to images (.tif type file) using the functions “videoreader” and “read”. The images were first preprocessed using a customized background subtraction algorithm. Briefly, a region of interest (ROI; blue polygon in **S1A Fig.**) was defined by the user in the first movie frame to create a mask for masked filtering. The function “roifill” smoothly interpolated into the ROI from the pixel values on the boundary by solving Laplace’s equation (**S1B Fig.**). We used this image as the background for each movie and subtracted it from the rest of the frames (**S1D Fig.**). All background subtracted grayscale images were then converted to binary images by using a function “im2bw” (**S1E Fig.**), and the images were “skeletonized” into a single backbone curve using the function “bwmorph” and the “thin” operation (**S1F Fig.**). The skeletonized pixel array was sorted into x and y coordinate vectors from the head to the tail of the fish. A smooth spline was fitted on this sorted fish backbone array and then discretized into 10 evenly spaced segments along the backbone arc length (**S1G Fig.**).

**Zebrafish analysis**

**Real space representation of fish backbone reconstructed using Eigenshapes.** The eigenshape $V_{k}\left(s_{j}\right)$ can be visualized in real space by translating the fish backbone angles into Cartesian coordinates *X_j_*, *Y_j_*. We define$\Delta\theta_{k}(s_{j},t_{i})$as the fish backbone angle reconstructed from the *k*^th^ eigenshape:

$$\Delta\theta_{k}(s_{j},t_{i})\equiv U_{k}(t_{i})S_{kk}V_{k}(s_{j})$$

The corresponding Cartesian coordinates *X_j_*, *Y_j_* are given by:

$$\begin{matrix} X_{j}=\sum_{j=1}^{n} \cos\left(\Delta\theta_{k}(s_{j},t_{i})\right) \\ Y_{j}=\sum_{j=1}^{n} \sin\left(\Delta\theta_{k}(s_{j},t_{i})\right) \end{matrix}$$

**S3 Fig.** displays the real-space representations of the 3 eigenshapes for each frame (*t_i_*) in a representative movie. The highlighted shapes correspond to three frames in which the contributions from one eigenshape is large (*V*_1_, blue; *V*_2_, red; *V*_3_, green). Eigenshapes *V*_1_, *V*_2_, and *V*_3_ correspond to shapes with one bend, two bends, and three bends, respectively.

**Time series alignment**. We quantified the behavioral variability in zebrafish free swimming by measuring the “distance” between trajectories. To this end, we first aligned the amplitudes *U*_1_, *U*_2_, *U*_3_ of each fish trajectory temporally, introducing a time shift and scaling the time axis, to maximize the overlap between them. Each trajectory $\alpha$ was assigned a “normalized time” $\tilde{t}^{\alpha}\equiv a_{\alpha}t+b_{\alpha}$ and the set of parameters {$a_{\alpha}$} and {$b_{\alpha}$} were optimized as described below.

The data were preprocessed before optimization by normalizing the time axis of *U*_1_, *U*_2_, *U*_3_ to the time period of cycle 1 of *U*_1_. Cycle 1 was defined as the time from the first to the third zero crossings of amplitude *U*_1_ as labeled in **S6A Fig. and Fig. 4B**. To find the optimal set of normalized times maximizing the overlap between trajectories, we minimized the following function:

$$F=\min\left(\frac{2}{m(n(n-1))}\sum_{\alpha=1}^{N} \sum_{\beta=1}^{\alpha} \sum_{i=1}^{m} ||S_{\alpha}(\tilde{t}_{i}^{\alpha})-S_{\beta}(\tilde{t}_{i}^{\beta})||^{2}-\lambda_{a}\sum_{\alpha=1}^{N} (a_{\alpha}-1)-\lambda_{b}\sum_{\alpha=1}^{N} (b_{\alpha}-0)\right)$$

over all frames *i* = 1… *m*, where

$$||S_{\alpha}(\tilde{t}_{i}^{\alpha})-S_{\beta}(\tilde{t}_{i}^{\beta})||^{2}\equiv[U_{1\alpha}(\tilde{t}_{i}^{\alpha})-U_{1\beta}(\tilde{t}_{i}^{\beta})]^{2}+[U_{2\alpha}(\tilde{t}_{i}^{\alpha})-U_{2\beta}(\tilde{t}_{i}^{\beta})]^{2}$$

and *N* is the number of trajectories, $\lambda_{a}$ is a Lagrange multiplier [1] that ensures the sum of the parameter $a_{\alpha}$ over all trajectories is 1 and $\lambda_{b}$ is a Lagrange multiplier that ensures the sum of the parameter $b_{\alpha}$ over all trajectories is 0. We minimized the least square difference over only two dimensions *U*_1_, *U*_2_ as we found that the variability in *U*_3_ to affect the minimization. The results of this optimization are shown in **S6** **Fig.** and **Fig. 4**.

**Dissimilarity matrix for MDS embedding.** We determined the “distance” or dissimilarity matrix $d_{\alpha\beta}^{2}$between trajectories from the Euclidean distance between pairs $\alpha, \beta$ of time-aligned spine bend angles $\Delta\theta_{\alpha}$ and $\Delta\theta_{\beta}$. We show here how the dissimilarity matrix can be expressed in terms of the amplitudes *U*_1_, *U*_2_, *U*_3_. The distance metric $d_{\alpha\beta}^{2}$ is given by:

$$d_{\alpha\beta}^{2} = \sum_{i,j} \left[\Delta\theta_{\alpha}\left(s_{j}, \tilde{t}_{i}^{\alpha}\right)- \Delta\theta_{\beta}\left(s_{j}, \tilde{t}_{i}^{\beta}\right)\right]^{2}$$

where $\tilde{t}_{\alpha}$ and $\tilde{t}_{\beta}$ are the normalized times for each trajectory. Substituting Eq. (1) for $\Delta\theta_{\alpha}$ and $\Delta\theta_{\beta}$ yields:

$$d_{\alpha\beta}^{2}= \sum_{i,j} \left\{ \sum_{k=1}^{n} S_{kk}V_{k}\left(s_{j}\right)\left[U_{k}^{\alpha}\left(\tilde{t}_{i}^{\alpha}\right) - U_{k}^{\beta}\left(\tilde{t}_{i}^{\beta}\right)\right]\right\}^{2}$$

which expands to:

$$d_{\alpha\beta}^{2}= \sum_{i,j} \left\{\sum_{k} \sum_{k'} S_{kk}V_{k}\left(s_{j}\right)S_{k'k'}V_{k'}\left(s_{j}\right)\left[U_{k}^{\alpha}\left(\tilde{t}_{i}^{\alpha}\right) - U_{k}^{\beta}\left(\tilde{t}_{i}^{\beta}\right)\right] \left[U_{k'}^{\alpha}\left(\tilde{t}_{i}^{\alpha}\right) - U_{k'}^{\beta}\left(\tilde{t}_{i}^{\beta}\right)\right] \right\}$$

From the orthonormality of the eigenshapes, i.e. $\sum_{j} V_{k}\left(s_{j}\right)V_{k'}\left(s_{j}\right)=\delta_{kk'}$, it follows that

$$d_{\alpha\beta}^{2}=\sum_{i} \sum_{k=1}^{n} S_{kk}^{2}\left[U_{k}^{\alpha}\left(\tilde{t}_{i}^{\alpha}\right) - U_{k}^{\beta}\left(\tilde{t}_{i}^{\beta}\right)\right]^{2}$$

which yields Eq. (2), when truncating the series to *n* = 3.

**Neural network model.** We used a customized version of a previously described neuro-kinematic model [2]. Our version of the neural network divides the fish backbone into a right and left half, each with 10 equal neural segments (*s_j_*) of neurons interconnected by synapses (see **S11A Fig.**). The right and left halves each receive a trigger signal $F_{osc}(s_{j},t_{i})$ in the form of a train of sharp pulses each of whose firing times *τ_f_*, amplitudes *a*, and segment (*s_j_*) to segment (*s_j_*_+1_) delays *d* are model parameters. The output of each neural segment along the backbone is convolved with a bi-exponential function representing the temporal response of the neuromuscular junction.

To determine the set of parameters that best reproduce the observed behavior, we optimized the model parameters against test data traces of a scoot and turn bout (**S11B, C Fig.**). We obtained the number of alternating left-right movements (or half-cycles) of the zebrafish in a particular bout, *n_c_*, by determining the number of zero crossings of the tail angle$\Delta\theta(s_{9},t_{i})$from the test experimental data. For example, **S11B, C Fig.** show a 3D plot of $\Delta\theta(s_{j},t_{i})$from a turn and a scoot, where *n_c_* = 7 and 8, respectively. Since young fish larvae swim with a decreasing tail beat frequency, we allowed the firing times $\tau_{f}^{l}$, amplitudes *a_l_*, and segment to segment delays *d_l_* to be functions of the half-cycle number *l* = 1, 2 ... *n_c_*.

The input parameters for optimization were the set $\{\tau_{f}^{l},a_{l},d_{l}\}$ with *l* = 1, 2 ... *n_c_* and the values of the stiffness function $W(s_{j})$from head (*j* = 0) to tail (*j* = 9). A genetic algorithm in MATLAB was used to minimize the sum of differences squared between the fish backbone angles$\Delta\theta^{neuro}(s_{j},t_{i})$ generated by the neural network and the fish backbone angles $\Delta\theta(s_{j},t_{i})$ obtained from the experimental data. The start values for the model parameters were obtained from experimental data. We measured the times *t_l_* at which the zero crossings occurred for the head and tail segments *s*_1_ and *s*_9_, respectively. These zeros crossings are the start value for input parameter spike firing time *τ_f_*. The starting value for the segment-to-segment delay *d_l_* of the *l*-th half-cycle was:

$$d_{l}=\frac{t_{l}\left(s_{9}\right)-t_{l}\left(s_{1}\right)}{9}$$

Finally, the starting value for the amplitude of the *l*-th half-cycle was:

$$a_{l}=\left\{\begin{matrix} 1 & l \text{even} \\ -1 & l \text{odd} \end{matrix}\right.$$

and the corresponding trigger signal was:

$$F_{osc}(s_{j},t_{i})=\left\{\begin{matrix} a_{l} & \tau_{f}^{l} \\ 0 & \text{otherwise} \end{matrix}\right.$$

The initial function $W(s_{j})$was chosen to be linear from head to tail. $W(s_{j})$ was optimized to minimize Eq. (S9). The stiffness function was optimized at the time of the maximum amplitude of the tail angle $\Delta\theta\left(s_{9},t_{i}\right)$ from a turn trajectory.

**Stability of trajectories produced by neural model**. We investigated the effect of noise on model parameters $\{\tau_{f}^{l},a_{l},d_{l}\}$ to evaluate the stability of trajectories$\Delta\theta^{neuro}(s_{j},t_{i})$ generated by the neural network. The impact of noise on these parameters was evaluated from the difference between $d_{\alpha\beta(neuro)}^{2}$ (see Eq. (S8)) generated by the neural network in the absence of noise and $d_{\alpha\beta(noise)}^{2}$obtained after adding noise. **S13A, C** **Fig.** displays $d_{\alpha\beta(noise)}^{2}-d_{\alpha\beta(neuro)}^{2}$ as a function of white Gaussian noise added at varying levels to model parameters (plotted as signal-to-noise ratio). We then embedded the trajectories generated by the neural network with noise in a behavioral space with the experimental data. **S13D-F** **Fig.** show simulated scoot (green colored symbols) and turn (red colored symbols) trajectories with signal-to-noise ratio = 1 (triangles), 6 (squares), and ∞ (diamonds) in *a*, *τ_f_*, and *d* (bottom left panel to bottom right panel). Trajectories with noise in spike firing time *τ_f_* are scattered the most in behavioral space, indicating a high sensitivity to noise in this parameter. We believe this is because noise disrupts the firing sequence of the right and left halves of the backbone, which leads to non-cyclic trajectories. In contrast, noise in the segment-to-segment delay has the least effect on behavior. Interestingly noise in spike amplitude *a* can convert a scoot trajectory into a turn and vice versa (see points with signal-to-noise ratio = 6).

**References**

1. Laratta, A. and F. Zironi, *Computation of Lagrange multipliers for linear least squares problems with equality constraints.* Computing, 2001. **67**(4): p. 335-350.

2. Hill, S.A., et al., *Neurokinematic modeling of complex swimming patterns of the larval zebrafish.* Neurocomputing, 2005. **65**: p. 61-68.

3. Burgess, H.A. and M. Granato, *Modulation of locomotor activity in larval zebrafish during light adaptation.* J Exp Biol, 2007. **210**(Pt 14): p. 2526-39.
